# Paroxetine-induced transient apoptosis with delayed neurogenesis triggers brain-preferential regeneration in developing zebrafish

**DOI:** 10.1371/journal.pone.0345903

**Published:** 2026-10-30

**Authors:** Tomomi Sato, Kaito Saito, Tsubasa Oyu, Kazuna Yamaguchi, Maiko Osawa, Yuki Shiko, Yohei Kawasaki, Tomohiro Kurisaki, Sachiko Tsuda, Takeshi Kajihara, Masabumi Nagashima

**Affiliations:** 1 Department of Anatomy, School of Medicine, Saitama Medical University, Moroyama-machi, Saitama, Japan; 2 Department of Obstetrics and Gynecology, School of Medicine, Saitama Medical University, Moroyama-machi, Saitama, Japan; 3 Division of Life Science, Graduate School of Science and Engineering, Saitama University, Saitama City, Saitama, Japan; 4 Department of Biostatics, School of Medicine, Saitama Medical University, Moroyama-machi, Saitama, Japan; University of Hyderabad, INDIA

## Abstract

Autism spectrum disorder (ASD) is a neurodevelopmental condition caused by a combination of genetic and environmental factors. Its cardinal symptoms include impaired social behavior involving the amygdala. Maternal use of antidepressants such as paroxetine during early pregnancy increases the risk of ASD in offspring. However, the underlying pathogenic mechanisms remain poorly understood. Herein, we demonstrate that early exposure of zebrafish embryos to paroxetine delays neurogenesis in the optic tectum and telencephalon, including a region corresponding to the mammalian amygdala. Paroxetine-treated embryos exhibit growth restriction characterized by small heads and short-body lengths resulting from transient apoptosis. This phenotype resembles the early-onset fetal growth restriction (FGR) associated with ASD. Interestingly, we observed regeneration of delayed neurogenesis in the reduced brain following paroxetine withdrawal. This regenerative process was accompanied by extended retinotectal projections, suggesting brain-preferential regeneration. Finally, we examined the social behavior of paroxetine-treated fish. Taken together, our findings suggest a biological mechanism linking early paroxetine exposure, a risk factor for ASD, to its pathogenesis.

## Introduction

Autism spectrum disorder (ASD) is a neurodevelopmental disorder diagnosed in childhood [1]. Its cardinal symptoms include impaired social behavior, communication deficits, and stereotyped repetitive behaviors [2]. To date, numerous genes have been identified as being associated with ASD, including those involved in synaptic function, transcriptional regulation, and brain development [1,3]. In addition, de novo mutations, advanced paternal age, and maternal infection or inflammation during pregnancy have been implicated as causative factors [3–5]. Thus, genetic and environmental factors contribute to the impaired brain development and function observed in ASD [4–6]. Maternal depression and anxiety during pregnancy are also associated with an increased risk of ASD in offspring [7]. Selective serotonin reuptake inhibitors (SSRIs), a commonly prescribed class of antidepressants, are administered to pregnant women based on risk–benefit considerations [8]. Although these drugs exert therapeutic effects on the maternal brain, they also cross the placenta and reach the fetus [9,10]. Several studies have suggested that maternal SSRI use during the first trimester of pregnancy increases the risk of ASD in offspring [11–14]. However, the underlying pathogenic mechanisms remain poorly understood.

Fetal growth restriction (FGR) is a common complication of pregnancy that can result from a variety of maternal, placental, and fetal factors [15]. Early-onset FGR is defined as symmetrical fetal growth restriction, resulting from a small head and body due to impaired fetal growth [16]. Its causative factors include genetic abnormalities, congenital malformations, intrauterine infections, and teratogenic effects [15]. Early-onset FGR has also been associated with ASD [17–19]. However, both the pathogenesis of ASD and the pathophysiological role of SSRIs in early-onset FGR remain poorly understood.

Serotonin (5-hydroxytryptamine [5-HT]) is a monoamine neurotransmitter involved in emotional disorders such as depression and anxiety through its modulation of synaptic transmission in the brain [20,21]. SSRIs exert their therapeutic effects by inhibiting 5-HT reuptake through the serotonin transporter (SERT), thereby maintaining extracellular 5-HT levels [22,23]. In addition, 5-HT plays an important role in adult neurogenesis by promoting the self-renewal of neural stem cells in the hippocampus [24]. Maternal 5-HT synthesized in the placenta of pregnant mice has been shown to transiently localize to the forebrain of early-stage embryos [25], raising the possibility that placenta-derived 5-HT regulates early brain development [26–28]. However, the effects of early SSRI exposure and 5-HT on embryonic brain development and postnatal behavior remain unclear. Elucidating these primary effects is essential for the early prediction and prevention of ASD risk. Although SSRI use during early pregnancy increases the risk of ASD, the primary cause remains unclear [11,13,14]. The involvement of multiple genetic and environmental factors further complicates a comprehensive understanding of the causes and consequences underlying ASD pathogenesis [1–4]. Therefore, elucidating the primary effects of SSRIs on early brain development and their subsequent impact on social behavior is important. To address this question, we used zebrafish as a model of human embryonic development because their early embryos are readily accessible in a living and intact state, allowing direct investigation of drug effects during development. Various zebrafish models of neurodevelopmental disorders have been established, suggesting evolutionarily conserved pathophysiological mechanisms between zebrafish and humans [29–31]. We focused on paroxetine because of its broad clinical use in affective disorders, including major depressive and anxiety disorders [32]. In this study, we used 5-HT as a comparator of paroxetine in order to segregate the direct from the indirect effects of paroxetine, that is, the SERT-inhibiting effect from the resulting increased extracellular 5-HT levels. We examined the effects of paroxetine and compared them with those of controls and 5-HT on morphology, neurogenesis, and neural projections during embryonic and larval stages, as well as on social behavior during juvenile stages.

## Materials and methods

### Zebrafish maintenance and strains

Wild-type and transgenic zebrafish were maintained at 28°C under a 14 h:10 h light/dark cycle as previously described [33] in an IWAKI Small Fish Breeding Unit LAbREED ITS. To obtain transgenic embryos, the wild-type strain Riken WT (RW) was crossed with the following transgenic lines: Tg(*elavl3:Kaede*) to visualize post-mitotic neurons [34]; TgBAC(*neurod1:EGFP*) to visualize differentiated neurons [35]; Tg(*pau4f1-hsp70l:GFP*) to visualize the development of post-mitotic excitatory neurons and retinal axons in the optic tectum [36]; Tg(*pau4f3:GAL4;UAS:mCherry*) to visualize retinal axons in the optic tectum [37]; and SAGFF120A;UAS:GFP to visualize neurons in the telencephalon [38]. All embryos and larvae were maintained at 28°C in E3 embryo medium (5 mM NaCl, 0.17 mM KCl, 0.33 mM MgSO_4_, 0.33 mM CaCl_2_), followed by E3 medium containing 0.003% 1-phenyl-2-thiourea (PTU) from approximately 8 hpf onward, with media refreshed daily. Embryos and larvae were manually dechorionated and used at the developmental stages specified for each experiment. For anesthesia, fish were treated with 0.04% tricaine (MS-222; ethyl m-aminobenzoate methanesulfonate, 14805−82, Nacalai Tesque), chilled on ice to minimize suffering, and subsequently sacrificed by fixation in 4% paraformaldehyde phosphate-bufferred solution (09154−14, Nacalai Tesque) at 4°C. All animal experiments were approved by the Animal Experimentation Committee of Saitama Medical University (Approval No. 4250) and conducted in accordance with the Regulations on Animal Experimentation of Saitama Medical University.

### Microinjection

Preparation and injection of antisense morpholino oligonucleotides (MOs) were performed as previously described [31]. Briefly, MOs were diluted in 1 × Danieau buffer (58 mM NaCl, 0.7 mM KCl, 0.4 mM MgSO_4_, 0.6 mM Ca(NO_3_)_2_, 5.0 mM HEPES, pH 7.6) and injected into 1- to 2-cell-stage embryos using a FemtoJet 5247 microinjector (Eppendorf). The sequences and concentrations of the MOs (Gene Tools, OR, USA) used in this study were as follows: slc6a4a-AMO (splice-blocking antisense MO, MO1-slc6a4a, ZDB-MRPHLNO-141031–1, ZIRC) [39], 5′-ACGCACTTACATGCACTTACACATA-3′, at 4.178 ng/nl; and slc6a4a-5mis (5mis; 5-bp mismatched control), 5′-ACCCACTTAGATCCAGTTAGACATA-3′, at 4.198 ng/nl.

### Drug treatments

Preparation of paroxetine and 5-HT solutions was performed as previously described [40]. Briefly, paroxetine (Mw 365.83, P1977, Tokyo Chemical Industry (TCI), Tokyo, Japan), fluoxetine (Mw 345.79, F0750, TCI), fluvoxamine (Mw 434.41, F0858, TCI), sertraline (Mw 342.69, 193−16191, Fujifilm Wako Chemicals, Osaka, Japan), and 5-HT (Mw 212.68, S0370, TCI) were dissolved in dimethyl sulfoxide (DMSO) (Nacalai Tesque, 13407−45, Kyoto, Japan) to prepare 10 mM stock solutions. These were subsequently diluted in E3 medium to final concentrations of 1–20 µM in 1% DMSO. Reported pharmacokinetic parameters of paroxetine indicate that the maximal blood concentration (C_max_) and time to maximal concentration (T_max_) are 1.93 ± 1.38 ng/mL and 4.61 ± 1.04 h, respectively, in healthy adults after a single oral dose of 10 mg paroxetine (Paxil Tablet, GSK). In our experiments, bath application of 1–20 µM paroxetine in E3 medium corresponded to 0.3748–7.4966 µg/mL in vitro, approximately 200–3900 times higher than the reported blood C_max_. Zebrafish embryos were dechorionated with forceps, immersed in the treatment solution from 26–27 hpf, and incubated at 28.5°C. Most embryos survived under these treatment conditions (Fig 2B), and surviving embryos were euthanized for subsequent analyses or following social behavioral testing.

### Immunohistochemistry

Whole-mount immunohistochemistry was performed as previously described [31]. Briefly, embryos were fixed in 4% paraformaldehyde/PBS and incubated with primary antibodies overnight at 4°C, followed by incubation with rabbit- or goat-specific secondary antibodies conjugated to Alexa Fluor 488 or 555 (Abcam, Cambridge, UK) together with DAPI (Thermo Fisher Scientific, MA, USA). The following primary antibodies were used: anti-active Caspase-3 (rabbit, 1:500 dilution, 559565, BD Pharmingen, NJ, USA); anti-phospho-histone H3 (pH3; rabbit, 1:500 dilution, 9701, Cell Signaling, MA, USA); anti-SOX2 (rabbit, 1:500 dilution, GTX124477, GeneTex, Hsinchu, Taiwan); anti-serotonin (goat, 1:500 dilution, ab66047, Abcam); anti-SLC6A4/5-HTT (rabbit, 1:100 dilution, PA5–49572, Thermo Fisher Scientific); and anti-Cyclin D1 [N1C3] (rabbit, 1:500 dilution, GTX108624, GeneTex). Following immunostaining, the PBS wash solution was gradually replaced with 30%, 50%, and finally 70% glycerol/PBS before imaging under a microscope.

### RNA extraction and RT-qPCR

Total RNA was extracted from whole embryos injected with MOs or treated with drugs using the miRNeasy Mini Kit according to the manufacturer’s instructions (Qiagen, Hilden, Germany). cDNA was synthesized from total RNA using SuperScript IV Reverse Transcriptase (Thermo Fisher Scientific, MA, USA) and oligo(dT) primers. Quantitative PCR (qPCR) was then performed using Power SYBR Green Master Mix (Thermo Fisher Scientific) and gene-specific primers. The primer sequences used in this study are as follows: 5′-ATCTCTCCCGCTGAAGAGTTTTATG-3′ (slc6a4a-forward), 5′-GCCAGAGGTCTTGACCCCCTTC-3′ (slc6a4a-reverse), 5′-CGAGTCTAGTTCGAGTCCGC-3′ (sox2-forward), 5′-CGGGCAGGTACATACTGATC-3′ (sox2-rerverse), 5′-TCTGGAGGACTGTAAGAGGTATGC-3′ (rpl13α-forward) [41], 5′-AGACGCACAATCTTGAGAGCA-3′ (rpl13α-reverse) [41]. qRT-PCR was performed using a PikoReal 96 Real-Time PCR System (Thermo Fisher Scientific, MA, USA). Relative expression levels of *slc6a4a* and *sox2* were normalized to *rpl13α* and calculated using the 2^−ΔΔCt^ method.

### Microscopy and imaging

Most individuals were observed in lateral views under a microscope unless otherwise noted. Each individual was mounted on a glass slide and positioned such that the left-right body axis was parallel to the optical axis under a stereoscopic microscope, as shown in S4 Fig. Whole-body images were obtained using a Leica MZ10F stereoscopic microscope equipped with an MC170 HD camera. Multicolor fluorescence images of the head and tail were acquired using a Zeiss LSM700 confocal laser-scanning microscope equipped with EC Plan-NEOFLUAR 20 × /NA 0.5 and LD Plan-NEOFLUAR 40 × /NA 0.6 objectives. Image acquisition and analysis were performed after validating the methods using different transgenic lines. To avoid variation in fluorescence intensity caused by image acquisition conditions, imaging parameters were initially determined using control individuals for each experiment, and all images were subsequently acquired under identical conditions. Each confocal image was generated by compiling approximately 30–40 optical sections at 2.0 µm intervals per hemisphere using sequential scanning for each frame with a frame size of 512 × 512 pixels and 8-bit depth. As a control, the mode of the fluorescence intensity frequency distribution within the ROI set in the hindbrain (S4B, C Fig), which was 20, was regarded as the background level and subtracted from the compiled images. The same image-processing procedure was applied to all images obtained in each experiment.

### Social behavioral analyses

A test arena (68 mm length × 39 mm width × 15 mm height) divided into three compartments (sibling and no-sibling areas, 10 mm in length on both sides; test area, 38 mm in length in the center) was used to assess social behavior in young fish (35–37 dpf). Swimming behavior in the test arena was recorded by capturing movies using a cMOS camera (Blackfly S BFS-U3-32S4M, Point Grey) for 5 min at 50 frames per second (fps). Each experiment consisted of the following five steps: (1) A fish was transferred to the test area and habituated for 5 min. (2) In the absence of siblings, swimming behavior was recorded for 5 min (no-sibling trial). (3) Siblings were transferred to the sibling area and habituated for 5 min. (4) In the presence of siblings, swimming behavior was recorded for 5 min (sibling trial). (5) Captured movies were converted to avi format using ImageJ software, and tracking analysis of the swimming fish was performed using UMA Tracker software (Release 15). Social behavior was analyzed by comparing (2) and (4) using the SPI, calculated as follows: SPI = (Sibling frames − No-sibling frames)/Total frames [42]. Sibling frames (blue) were defined as frames in which the fish was located in the half of the test area adjacent to the sibling area, whereas no-sibling frames (red) were defined as frames in which the fish was located in the opposite half of the test area. SPI values ranged from −1–1 [42]. The SPI difference was considered positive when the SPI in the sibling trial was greater than that in the no-sibling trial and negative when the SPI in the sibling trial was lower than that in the no-sibling trial. The difference in total distance was defined as the value in the sibling trial minus that in the no-sibling trial.

### Quantification and statistical analyses

Body length, head area, and Kaede-, GFP-, or mCherry-positive areas and intensities were measured using Adobe Photoshop (2023, Ver. 24.4.1) and NIH ImageJ (Ver. 1.53). Confocal stacks were then converted into 2D images using sum-intensity projection. To eliminate ectopic signals derived from noise, regions exceeding a defined threshold and comprising contiguous pixels with an area of 3 pixels or greater were considered foreground. These regions were defined as fluorescence-positive areas, and both the area and mean fluorescence intensity were measured. The average of the control values was calculated, and each individual value was then expressed relative to this average. Thus, the average of the controls was set to 1.0 for every experiment. For statistical analysis of two or more groups, two-tailed, unpaired, nonparametric Mann–Whitney U tests or Kruskal–Wallis tests with Dunn’s multiple-comparison tests were performed, unless otherwise noted, using GraphPad Prism 9 (Ver. 9.5.1). A permutation test was used to assess differences in SPI between sibling and no-sibling trials. For each comparison, individual SPI differences were randomly permuted between groups, and the test statistic was recalculated for each permutation. A total of 1,000 permutations were performed to generate a null distribution. All statistical analyses were performed using SAS version 9.4 for Windows (SAS Institute, Cary, NC, USA).

## Results

### Amino acids interacting with paroxetine are conserved between human and zebrafish SERT sequences

To investigate the effects of paroxetine on brain development in zebrafish embryos, we first examined whether the amino acid sequence of the SERT, the target of paroxetine, is conserved between humans and zebrafish (Fig 1). The three-dimensional structure of the paroxetine-binding site and its interacting amino acids in human SERT has been well characterized by X-ray crystallography (Fig 1A) [43,44]. The overall amino acid sequence showed 70% identity between the two species (Fig 1B). Notably, all amino acids that interact with paroxetine at the central binding site of human SERT were identical between humans and zebrafish (Fig 1A, B). We next examined the expression of zebrafish SERT proteins by immunohistochemistry (Fig 1C and S1 Fig). At 30 h post-fertilization (hpf), when pet1-positive serotonergic neurons develop in the raphe nuclei, SERT proteins were weakly but broadly expressed throughout the brain (S1 Fig) [45]. We further found that SERT proteins were localized to the apical surfaces of radial glial cells in the ventricular zone at 24 hpf, implying that SERT plays a role in neural stem/progenitor cells.

**Fig 1:**
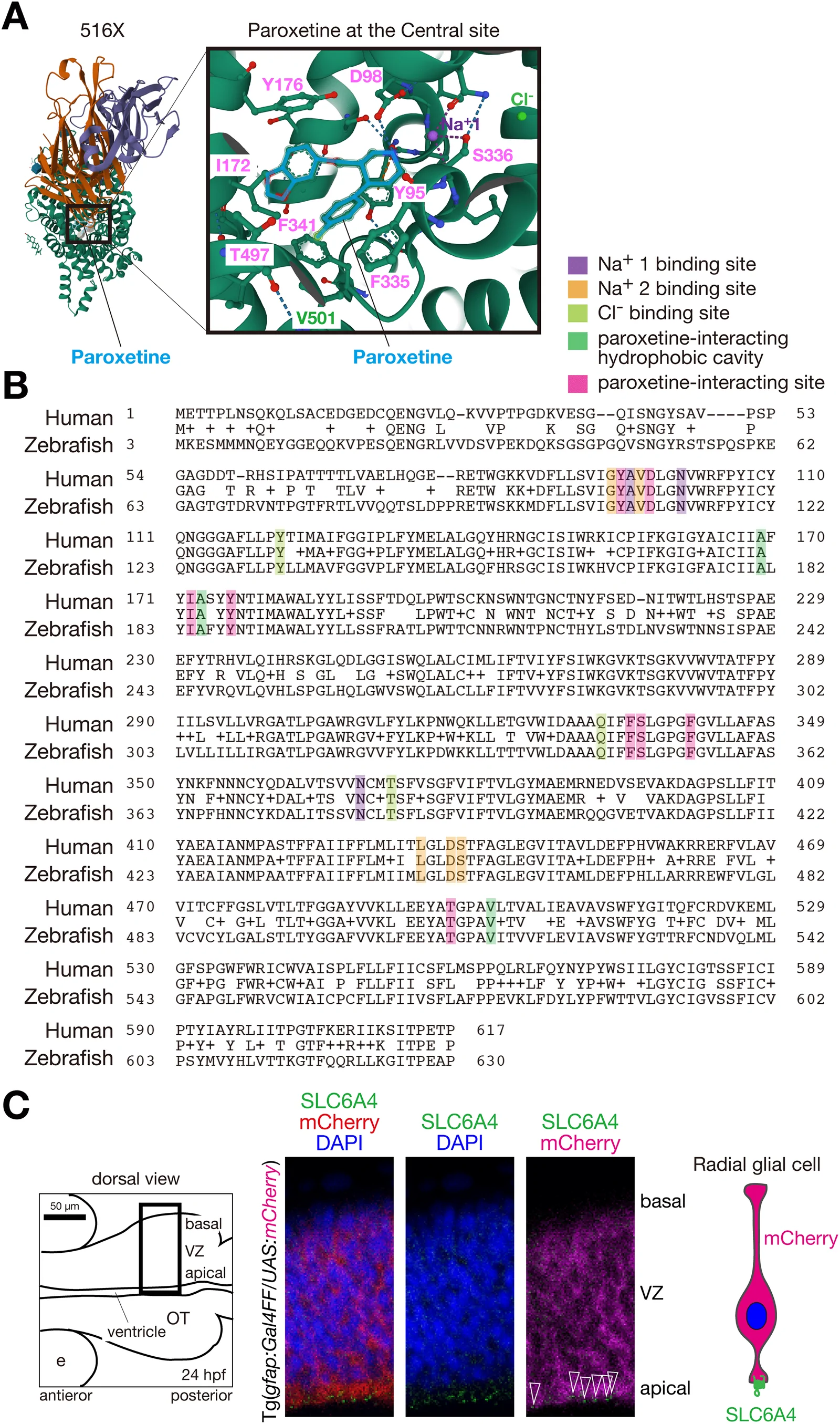
Amino acids interacting with paroxetine are conserved between human and zebrafish SERT sequences. (A) X-ray structure of the ts3 human SERT complexed with paroxetine at the central site (5I6X, RCSB Protein Data Bank). Amino acids interacting with paroxetine are indicated in pink, and an amino acid forming the hydrophobic cavity is indicated in green. Paroxetine is shown in light blue. (B) Alignment of the amino acid sequences of human and zebrafish SERT (i.e., P31645 and NP001035061). Amino acids interacting with paroxetine at the central site are completely conserved between human and zebrafish SERT. (C) SLC6A4 (SERT) proteins localized to the apical surface of the ventricular zone in the optic tectum (green, open arrowheads). A schematic of the embryonic brain in dorsal view is shown on the left. The corresponding region shown at higher magnification on the right is indicated by the bold rectangle. A schematic of an RFP-expressing radial glial cell and SLC6A4 localization is shown on the right.

### Early transient paroxetine exposure generates embryos with short-body and small-head phenotypes that are regenerated at later larval stages

To clarify the effects of paroxetine on embryonic development, we treated embryos with paroxetine or the effector 5-HT between 26 and 50 hpf, a period that roughly corresponds to the first trimester of human development (Fig 2A) [46]. To determine appropriate subthreshold concentrations for treatment, embryos were exposed to different concentrations of paroxetine and 5-HT, and survival rates were calculated. Neither compound affected survival at concentrations below 20 µM (Fig 2B). We then measured head-to-tail body length and head area in paroxetine-treated embryos as developmental parameters corresponding to fetal growth, estimated by crown-rump length during weeks 8–11 of the first trimester in humans [47]. First, we characterized normal development in wild-type embryos. In these experiments, body length and head area increased progressively and were significantly greater at later developmental stages than at 50 hpf (S2 Fig). Embryos treated with 10 µM paroxetine exhibited growth restriction, showing slight but significant reductions in both body length and head area at 50 hpf, whereas no significant reductions were observed in embryos treated with 1 µM paroxetine (Fig 2C-E). This phenotype closely resembled early-onset FGR during the first trimester of human pregnancy [16]. In contrast, treatment with either 1 or 10 µM 5-HT did not affect body length or head area, indicating that the observed embryonic growth restriction is a paroxetine-specific effect (Fig 2C-E). Embryos treated with 1 µM paroxetine for 2 days exhibited significantly shorter body lengths and smaller head areas at 73 hpf (S3 Fig). In addition, treatment with 10-µM paroxetine for 1 day produced a similar effect on larval morphology at 3 dpf, suggesting that transient exposure during early developmental stages is sufficient for paroxetine to exert its growth-inhibitory effects (S3 Fig). We therefore examined the effects of transient early paroxetine exposure on later development. At 5 dpf, larvae treated with 10 µM paroxetine during 26–50 hpf exhibited significant reductions in body length and head area relative to controls (S4 Fig). Interestingly, despite persistent reductions in body length, paroxetine-treated larvae showed regrowth of head area by 7 dpf relative to controls (Fig 2F-I). These observations suggest that brain-preferential regeneration occurs between 5 and 7 dpf.

**Fig 2:**
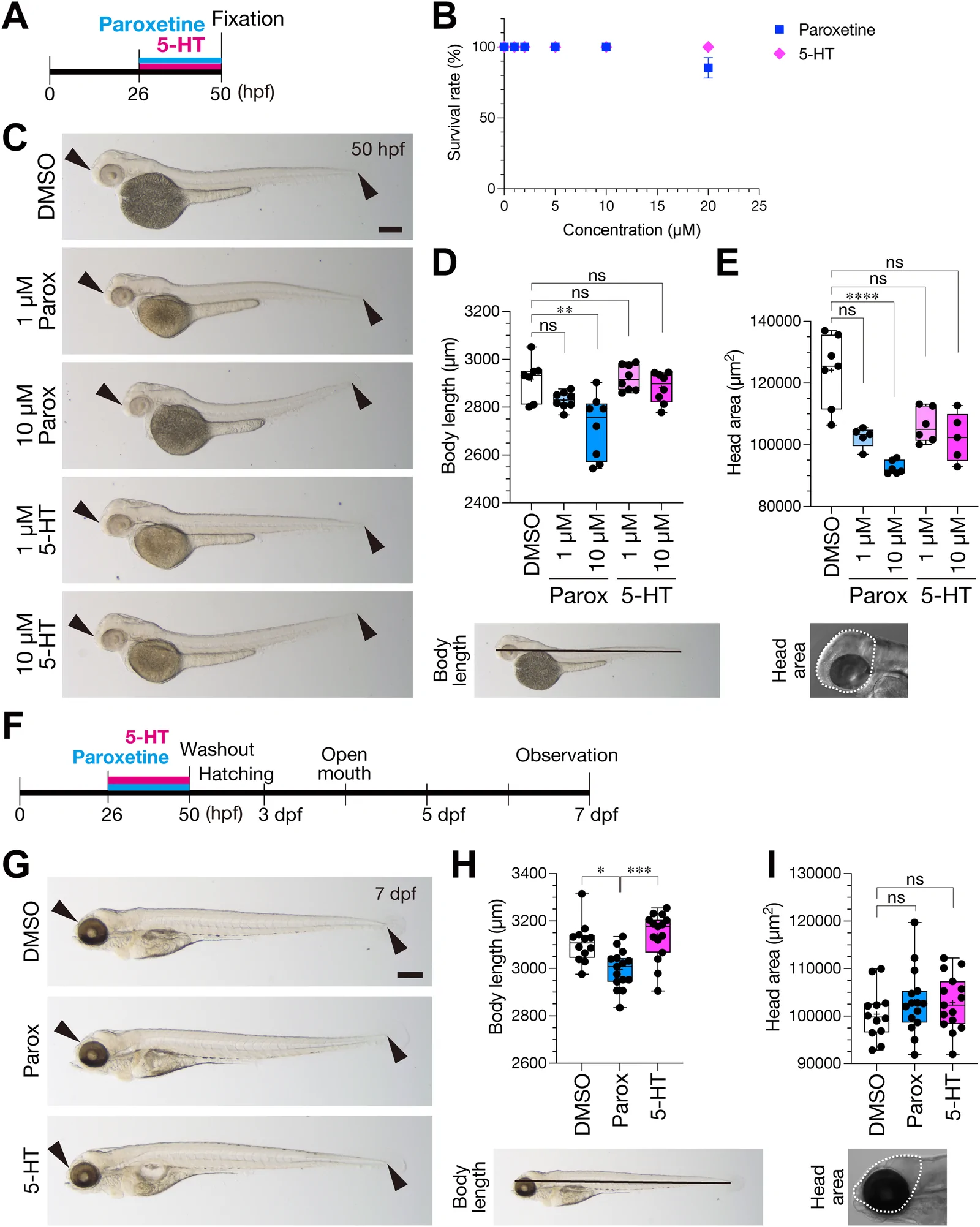
Early paroxetine induces embryonic growth restriction followed by preferential regrowth of the head during larval stages. (A) Schematic of the treatment time course. (B) Dose-response curves of survival rates in embryos treated with paroxetine (blue) or 5-HT (magenta). (C) Lateral views of 50-hpf zebrafish embryos treated with paroxetine (parox), 5-HT, or the control DMSO. (D, E) Quantification of body length (D) and head area (E) in the treated embryos. Representative images indicating the quantified body length (black line, D) and head area (dotted line, E) are shown below. (F) Schematic of the treatment schedule and major developmental events in zebrafish. (G) Lateral views of 7-dpf larvae treated with parox, 5-HT, or DMSO. (H, I) Quantification of body length (H) and head area (I) in the treated larvae. Representative images indicating the quantified body length (black line, H) and head area (dotted line, I) are shown below. Data are presented as box-and-whiskers plots. Whisters indicate the minimum and the maximum, the line and + in the box represent the median and the mean, respectively. *p < 0.05, **p < 0.01, ***p < 0.001, ****p < 0.0001; ns, not significant. Kruskal-Wallis with Dunn’s multiple comparisons tests. DMSO, dimethylsulfoxide; Parox, paroxetine.

### Paroxetine, but not 5-HT, transiently induces local apoptosis in the head and tail tip

To elucidate the mechanisms underlying the different effects of paroxetine and 5-HT on embryonic growth and development, we examined apoptosis at 31 hpf, 5 h after treatment with paroxetine or 5-HT, by immunostaining with an antibody against active Caspase-3 (Fig 3A, B). We found a significant increase in active Caspase-3 immunofluorescence throughout the brains of paroxetine-treated embryos relative to control dimethyl sulfoxide (DMSO)-treated embryos (Fig 3B, D, parox). Paroxetine-induced apoptosis increased in a dose-dependent manner (Fig 3E). In contrast, no such increase was detected in 5-HT-treated embryos (Fig 3B, D, 5-HT). This finding is consistent with our observation that 5-HT had no effect on head area (Fig 2C, E). Co-treatment with paroxetine and 5-HT also increased apoptosis in the brain, suggesting that apoptosis is induced by paroxetine-mediated inhibition of 5-HT uptake through SERT rather than by extracellular 5-HT itself (Fig 3F). Moreover, in addition to the widespread apoptosis observed in the brain, apoptosis was locally induced in the tail tip of paroxetine-treated embryos in an almost all-or-none manner (Fig 3C, G). Consistent with the phenotype of paroxetine-treated embryos, *slc6a4a* (*serta*)-knockdown embryos also exhibited reduced body length and increased apoptosis in the brain (S1 Fig). These results indicate that the increase in apoptosis is primarily caused by reduced intracellular 5-HT transport resulting from inhibition or knockdown of SERT. Thus, local apoptosis in the brain and tail tip corresponded with the growth restriction observed in paroxetine-treated embryos, including reduced head area and body length (Fig 2C-E). We next examined whether apoptosis persisted in the brains of paroxetine-treated embryos at 48 hpf. At this stage, brain-wide Caspase-3-positive signals were decreased in paroxetine-treated embryos. Indeed, signal intensities were significantly reduced in both the optic tecta (OT) and telencephalons (T), indicating that the apoptosis acutely induced after treatment was subsequently suppressed at later developmental stages (Fig 3H-J).

**Fig 3:**
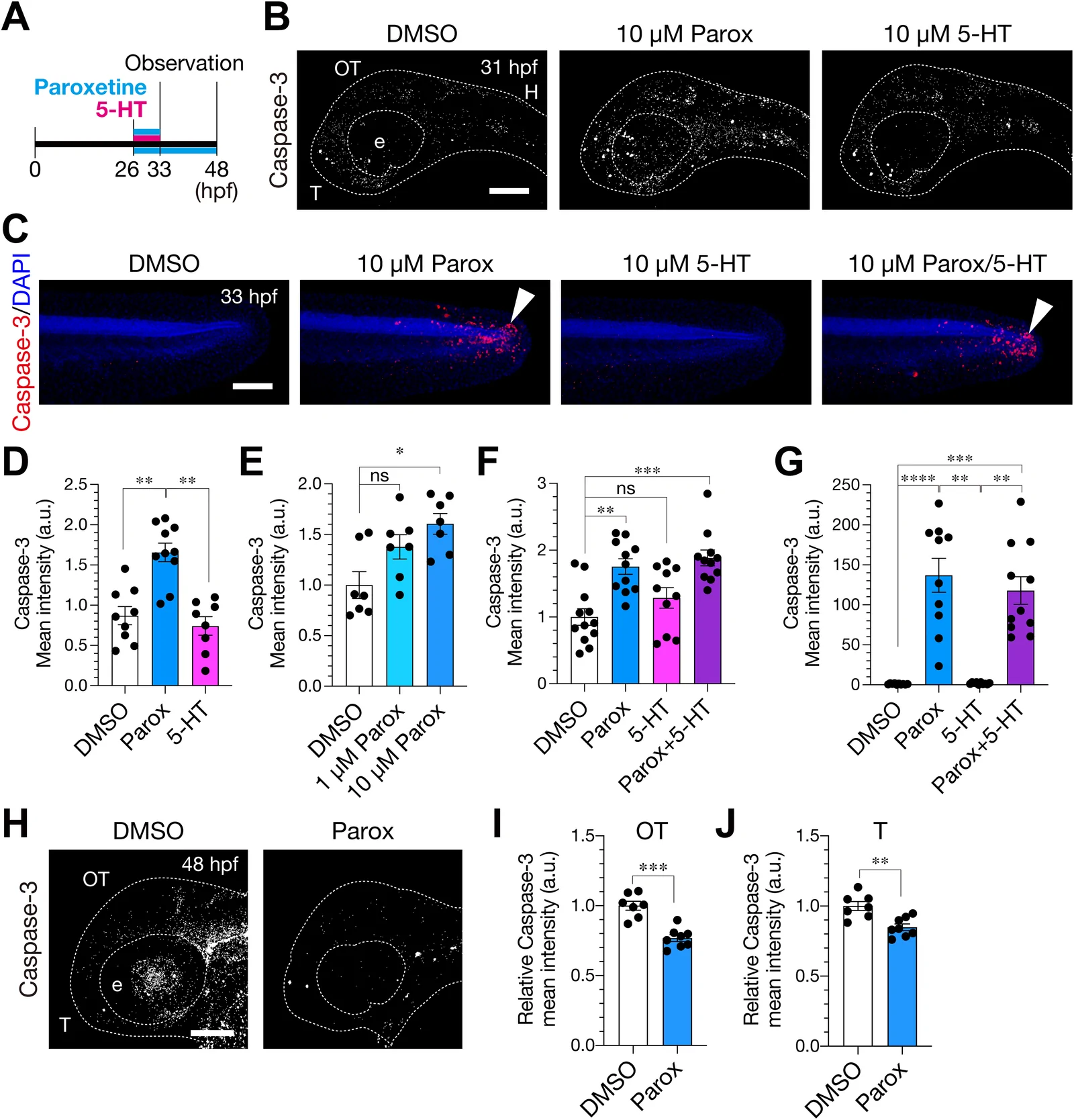
Paroxetine transiently induces local apoptosis in the brains and tail tips of treated embryos. (A) Schematic of the treatment time course. (B) Lateral views of the heads of 31-hpf embryos treated with DMSO, parox, or 5-HT and immunostained for active Caspase-3 (white puncta). Images were binarized above a threshold. Dotted lines indicate morphological outlines. (C) Lateral views of the tail tips of 33-hpf embryos treated with DMSO, parox, or 5-HT and immunostained for active Caspase-3. Anterior, left; posterior, right. (D-G) Quantification of the mean intensities of active Caspase-3 signals in the heads (D-F) and tail tips (G). (H) Lateral views of DMSO- or parox-treated embryos immunostained for active Caspase-3 (white puncta) at 48 hpf. Images were binarized above a threshold. Dotted lines indicate morphological outlines. (I, J) Quantification of the mean intensities of active Caspase-3 signals in the optic tecta (I) and telencephalons (J) of treated embryos. Scale bars: 100 µm (B, C, H). Data are presented as mean ± SEM. *p < 0.05, **p < 0.01, ****p < 0.0001; ns, not significant. Kruskal–Wallis with Dunn’s multiple-comparisons tests. Parox, paroxetine; 5-HT, serotonin; DMSO, dimethyl sulfoxide; hpf, hours post-fertilization; SEM, standard error of the mean; OT, optic tectum; T, telencephalon.

### Paroxetine decreases post-mitotic neurons while increasing proliferation of neural progenitor cells in the optic tectum

We next investigated the effect of paroxetine on neurogenesis in the optic tectum, a well-characterized visuomotor center in the zebrafish brain with respect to neural circuit development and function [31,48–51]. For this purpose, we used the transgenic lines TgBAC(*neurod1:EGFP*), which expresses enhanced green fluorescent protein (EGFP) in differentiated neurons [35], and Tg(*elavl3:Kaede*), which expresses Kaede under the control of the pan-neuronal promoter *elavl3* (*huC*) [34] (Fig 4A and S4 Fig). To evaluate the effects of paroxetine and other SSRIs on neurogenesis in intact embryos, we measured fluorescence-positive areas and fluorescence intensity as simple indicators of the number of post-mitotic neurons expressing fluorescent proteins such as EGFP or Kaede. We previously demonstrated that GFP intensity in post-mitotic neurons increases with developmental progression because it reflects both the number of neurons and the time elapsed since their differentiation [31]. The imaging and relative quantification procedures are described in S4 Fig and the Materials and Methods. We found that treatment with paroxetine during 26–50 hpf significantly reduced EGFP and Kaede fluorescence in post-mitotic neurons within both the optic tectum and telencephalon relative to controls (Fig 4A-C and S4 Fig). Consistently, the number of EGFP-positive neurons (intensity > 70) in the optic tectum was significantly decreased in paroxetine-treated embryos at 50 hpf (Fig 4D), suggesting that increased apoptosis induced by paroxetine reduced the number of post-mitotic neurons (Fig 3B, D–F). Treatment with other SSRIs, including fluoxetine, fluvoxamine, and sertraline, similarly decreased the number of post-mitotic neurons in TgBAC(*neurod1:EGFP*) embryos, indicating that this effect is shared among SSRIs (Fig 4G and S4 Fig). Immunohistochemical analysis of CyclinD1 further revealed a significant reduction in protein expression in the optic tectum of SSRI-treated embryos (Fig 4H and S4 Fig). We then examined the effects of elevated extracellular 5-HT by treating embryos with 5-HT during the same developmental period. Similar to paroxetine treatment, EGFP fluorescence was significantly reduced in the optic tectum of 5-HT-treated embryos relative to controls (Fig 4E, F), indicating that the effect of paroxetine on post-mitotic neurons can be recapitulated by 5-HT even in the absence of apoptosis induction (Fig 3B, D, F). This observation implied that reduced neurogenesis can potentially result from expansion of neural stem/progenitor cells.

**Fig 4:**
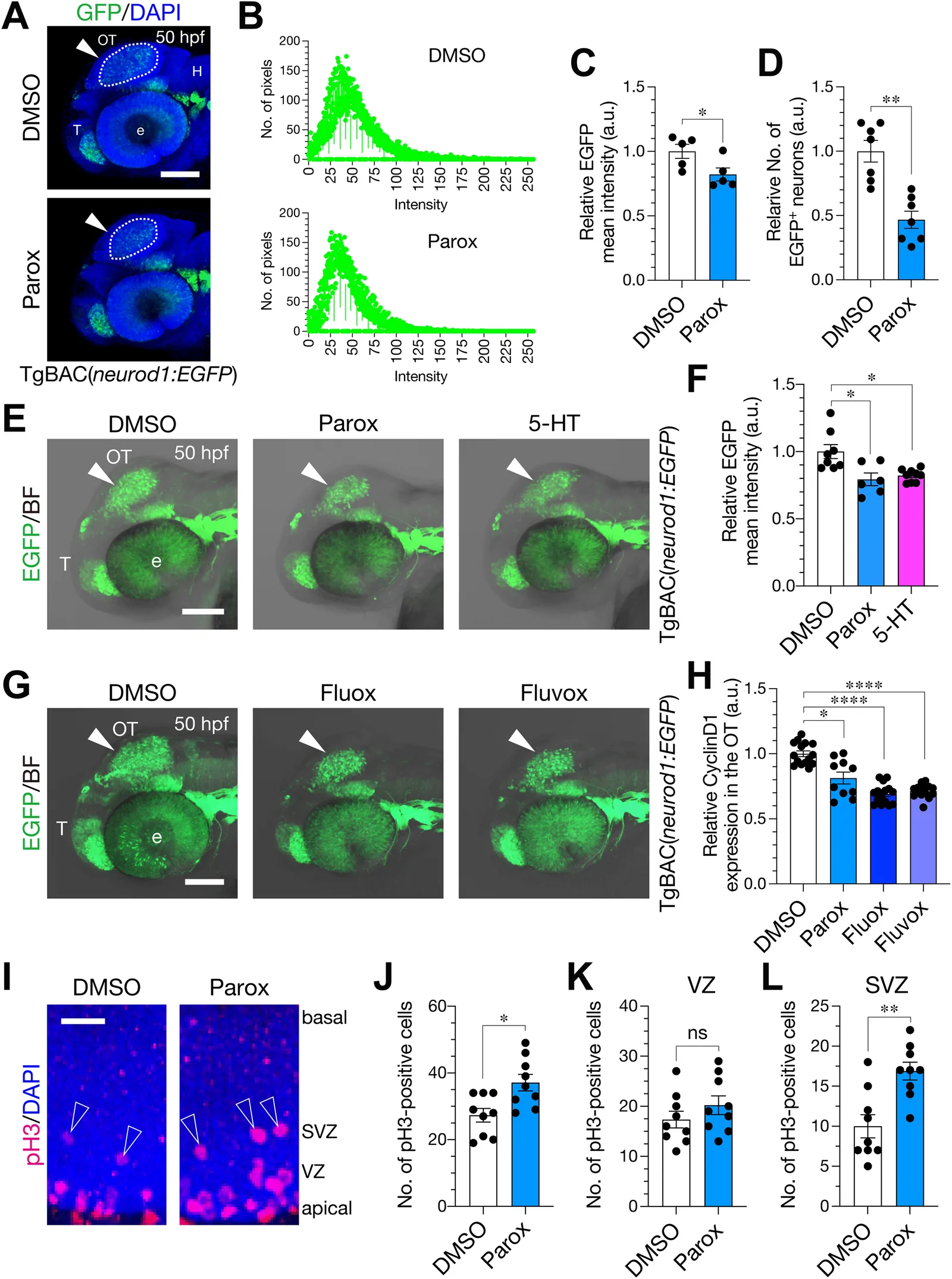
Paroxetine decreases post-mitotic neurons and promotes the proliferation of neural progenitor cells in the optic tectum. (A) Representative lateral views of 50-hpf TgBAC(*neurod1:EGFP*) embryos treated with paroxetine or the DMSO control. The EGFP-positive region in the optic tectum used for quantification is indicated by a dotted ellipse (arrowhead). (B) Intensity frequency distributions of the EGFP-positive region in the optic tectum of DMSO-treated (top) and paroxetine-treated (bottom) embryos (DMSO, 24.38 ± 1.359 pixels; paroxetine, 21.30 ± 0.882 pixels; n = 5 each). (C) Quantification of the mean EGFP intensity in the optic tectum (dotted ellipse indicated by arrowheads in A). Unpaired *t* test. (D) Quantification of the number of EGFP-positive neurons with intensities above 70 in the optic tectum. (E) Lateral views of 50-hpf TgBAC(*neurod1:EGFP*) embryos treated with paroxetine, 5-HT, or the control DMSO. (F) Quantification of the mean EGFP intensity in the optic tecta (arrowheads in E). Kruskal–Wallis with Dunn’s multiple-comparisons tests. (G) Lateral views of 50-hpf TgBAC(*neurod1:EGFP*) embryos treated with SSRIs (fluoxetine and fluvoxamine) or the control. (H) Quantification of CyclinD1 protein expression in the optic tectum of embryos treated with paroxetine, fluoxetine, fluvoxamine, or the control. (I) High-magnification images of the optic tecta of zebrafish embryos treated with DMSO or paroxetine and immunostained for pH3 (red), with DAPI (blue) counterstaining of nuclei. Open arrowheads indicate pH3-positive cells in the SVZ. (J-L) Quantification of the number of pH3-positive cells in the optic tecta (J), ventricular zones (K), and subventricular zones (L). Scale bars: 100 µm (A, E, G); 25 µm (I). All data are presented as mean ± SEM. *p < 0.05, **p < 0.01, ***p < 0.001, ****p < 0.0001; ns, not significant. Mann–Whitney *U* test. 5-HT, serotonin; DAPI, 4′,6-diamidino-2-phenylindole; DMSO, dimethyl sulfoxide; e, eye; EGFP, enhanced green fluorescent protein; H, hindbrain; hpf, hours post-fertilization; OT, optic tectum; parox, paroxetine; fluox, fluoxetine; fluvox, fluvoxamine; pH3, phospho-histone H3; SEM, standard error of the mean; SVZ, subventricular zone; T, telencephalon; VZ, ventricular zone.

In adult neurogenesis in rodents and humans, SSRIs are known to promote the proliferation of neural stem cells [24,52]. We therefore examined the proliferation of neural stem/progenitor cells in the optic tectum by immunostaining with an anti-phospho-histone H3 (pH3) antibody, a marker of mitotic cells [31]. We found that paroxetine-treated embryos exhibited a significant increase in the number of pH3-positive mitotic cells at 50 hpf (Fig 4I, J). This increase was selectively observed in the subventricular zone (SVZ), where intermediate progenitor cells undergo cell division during neurogenesis and proliferation (Fig 4I, K, L) [53]. Moreover, the expression of SOX2, a high-mobility group (HMG)-box transcription factor that promotes self-renewal in proliferating neural stem/progenitor cells, was enhanced in paroxetine-treated embryonic brains. In the same samples, we observed a concomitant decrease in Kaede-expressing neurons at 48 hpf, although an increase in *sox2* mRNA expression was not detected by RT-qPCR using whole embryos (S4 Fig) [40]. Taken together, these results suggest that paroxetine secondarily promotes the proliferation of neural progenitor cells following apoptosis and consequently impairs neurogenesis in the optic tectum.

### Development of retinotectal projections is impaired by early transient paroxetine exposure and regenerated with extended retinal axons

To investigate whether the reduction in neuronal number in paroxetine-treated embryos affects subsequent neural circuit formation, we examined retinotectal projections. We used the transgenic line Tg(*pou4f1-hsp70l:GFP*), which expresses GFP in excitatory tectal neurons and retinal ganglion cells (RGCs), and the line Tg(*pou4f3:GAL4;UAS:mCherry*), which expresses mCherry in RGCs [34,35]. In wild-type larvae, GFP- and mCherry-positive areas in the optic tectum increased progressively and significantly during development relative to those at 72 hpf (S2 Fig). During the same developmental period, however, the fluorescence intensities of GFP- and mCherry-positive regions significantly decreased between 5 and 7 dpf, when activity-dependent competition and subsequent synapse formation or elimination occur in retinal axons and tectal dendrites, respectively (S2 Fig) [48,49]. Treatment with 1 µM paroxetine for 2 days significantly reduced both the area and fluorescence intensity of GFP- and mCherry-expressing regions in the optic tectum at 73 hpf (Fig 5A-E). Similarly, 1-day treatment with 10-µM paroxetine produced a comparable effect on the GFP-expressing region (S3 Fig). These findings indicate that transient early exposure to paroxetine suppresses development of retinotectal projections, consistent with its growth-inhibitory effects on larval morphology (S3 Fig). We therefore examined the consequences of transient early paroxetine exposure on later retinotectal development. Consistent with regrowth of head area at 7 dpf (Fig 2G, I), paroxetine-treated larvae exhibited regeneration of both the area and fluorescence intensity of the GFP-expressing region, as well as regeneration of mCherry fluorescence intensity in the optic tectum to control levels (Fig 5F-H, J). Furthermore, the mCherry-positive area was significantly expanded in paroxetine-treated larvae relative to controls (Fig 5I, parox). This expansion was also observed in 5-HT-treated larvae (Fig 5I, 5-HT), suggesting that transient early exposure to paroxetine promotes extension of retinal axons through a 5-HT-dependent mechanism during later stages of neurodevelopment.

**Fig 5:**
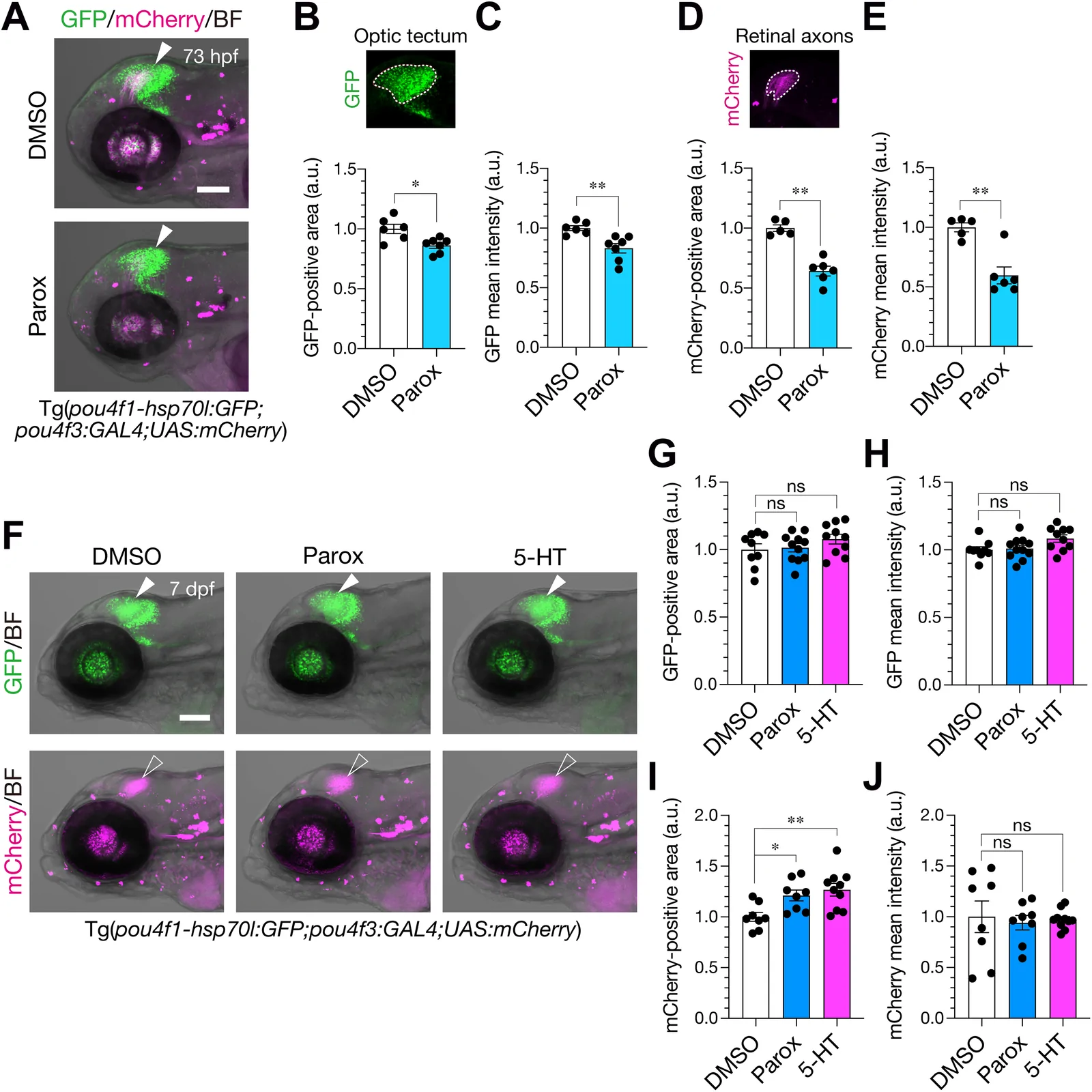
Early transient paroxetine inhibits development of the retinotectal projection, followed by brain-preferential regeneration. (A) Lateral views of the heads of 73-hpf Tg(*pou4f1-hsp70l:GFP; pou4f3:GAL4;UAS:mCherry*) embryos treated with DMSO or 1 µM paroxetine for 2 days from 26 hpf. GFP (green) is expressed in tectal neurons and retinal axons, whereas mCherry (magenta) is expressed in retinal axons. (B) Quantification of GFP-positive areas. (C) Mean GFP intensities in the optic tecta of the transgenic embryos. (D) Quantification of mCherry-positive areas. (E) Mean mCherry intensities in the optic tecta of the transgenic embryos. Representative images indicating the quantified area (dotted line) in the optic tectum are shown in the upper insets of (B) and (D). Mann–Whitney *U* test. (F) Lateral views of the heads of 7-dpf Tg(*pou4f1-hsp70l:GFP;pou4f3:GAL4;UAS:mCherry*) larvae treated with paroxetine, 5-HT, or the control DMSO. Filled arrowheads indicate optic tecta, and open arrowheads indicate retinal axons. (G-J) Quantification of GFP-positive areas (G), mean GFP intensities (H), mCherry-positive areas (I), and mean mCherry intensities (J) in the optic tecta of the transgenic larvae. Kruskal–Wallis with Dunn’s multiple-comparisons tests. Scale bar, 100 µm. Data are presented as mean ± SEM. *p < 0.05, **p < 0.01; ns, not significant. 5-HT, serotonin; DMSO, dimethyl sulfoxide; GFP, green fluorescent protein; hpf, hours post-fertilization; parox, paroxetine; SEM, standard error of the mean.

### Early paroxetine negatively impacts neurogenesis in the telencephalon

Next, we investigated whether early paroxetine treatment affects development of the telencephalon, which includes a region corresponding to the mammalian amygdala [29]. The amygdala plays a critical role in social behavior, and its dysfunction is associated with the impaired social behavior characteristic of ASD [2]. To assess the effects of paroxetine, we used the SAGFF120A gene-trap line, which locally expresses Gal4FF in a population of telencephalic neurons essential for active avoidance fear conditioning [38]. Similar to the findings in the optic tectum, fluorescence intensity in the GFP-expressing region of the telencephalon was significantly reduced in both paroxetine- and 5-HT-treated embryos relative to controls at 76 hpf (parox, *p* = 0.0279; 5-HT, *p* = 0.0250; Fig 6B-E). At 48 hpf, however, the decrease was significant in 5-HT-treated embryos but not in paroxetine-treated embryos (parox, *p* = 0.3344; 5-HT, *p* = 0.0006; S6 Fig). Consistent with the regenerative effects observed in the optic tectum, GFP fluorescence in the telencephalon recovered by 7 dpf following washout of paroxetine or 5-HT at 2 dpf (parox, *p* = 0.1419; 5-HT, *p* = 0.6623; Fig 6C-F). This regeneration occurred concomitantly with regrowth of head area and was consistent with the regenerative changes observed in the larval optic tectum (Figs 2I, 5F, H, J, and S6 Fig).

**Fig 6:**
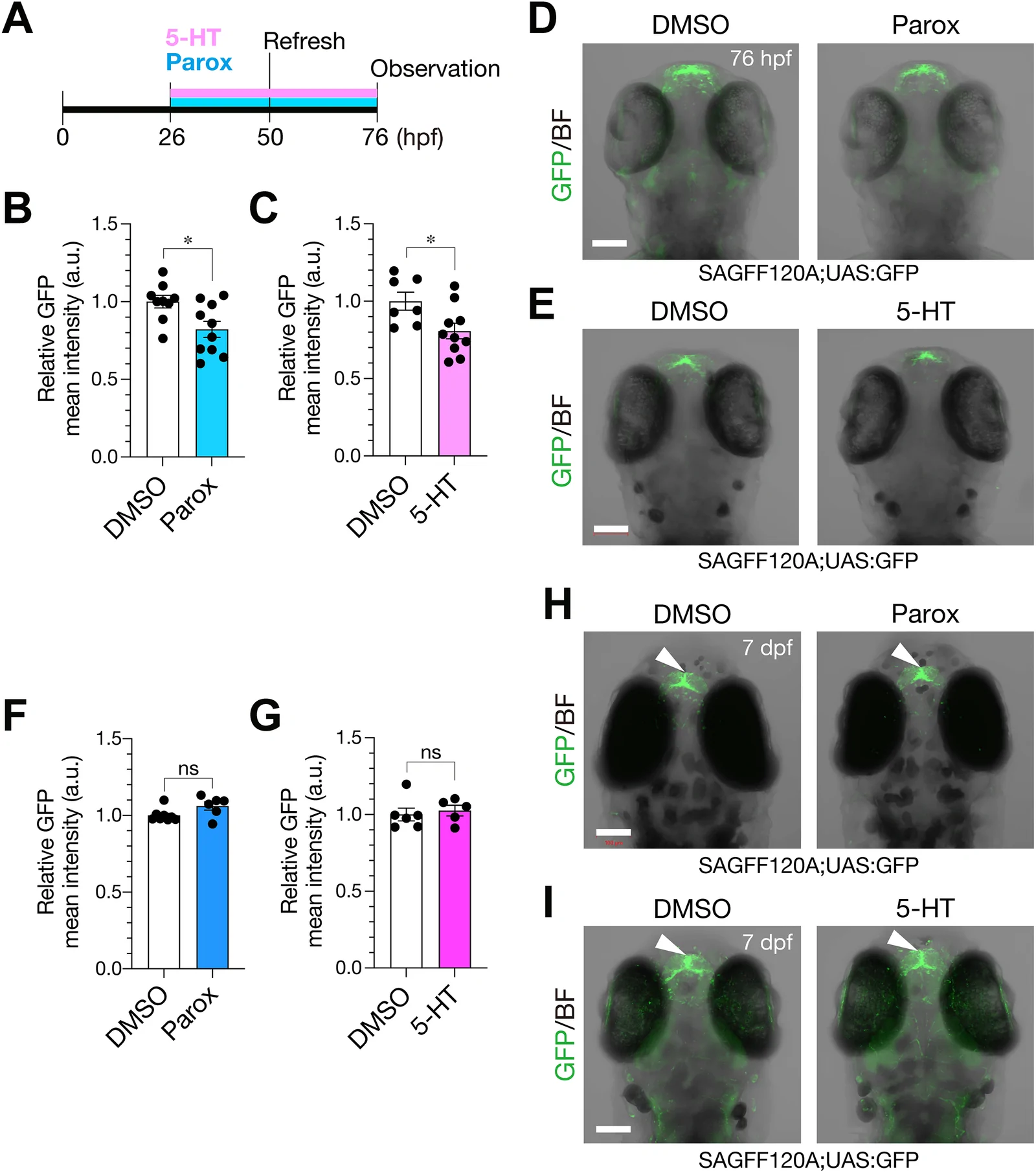
Early transient paroxetine suppresses neurogenesis, followed by regeneration in the telencephalon. (A) Schematic diagram of treatment with 1 µM paroxetine. (B, C) Quantification of GFP intensity in the telencephalon of paroxetine-treated (B) and 5-HT-treated (C) larvae. (D, E) Dorsal views of 76-hpf SAGFF120A;UAS:GFP larvae treated with paroxetine (D), 5-HT (E), or the control DMSO. (F, G) Quantification of mean GFP intensities in the telencephalons of paroxetine-treated (F) and 5-HT-treated (G) larvae. *p* = 0.1419 (F), *p* = 0.6623 (G), Mann–Whitney *U* test. (H, I) Dorsal views of 7-dpf SAGFF120A;UAS:GFP larvae treated with paroxetine (H), 5-HT (I), or the control DMSO. Scale bar, 100 µm. Data are presented as mean ± SEM. *p < 0.05, ***p < 0.001; ns, not significant. 5-HT, serotonin; DMSO, dimethyl sulfoxide; GFP, green fluorescent protein; hpf, hours post-fertilization; parox, paroxetine; SEM, standard error of the mean.

Finally, we investigated whether these neurodevelopmental effects of transient early paroxetine exposure influence social behavior during the juvenile stage. This analysis was facilitated by the absence of obvious differences in food intake or feeding behavior among paroxetine-treated, 5-HT-treated, and control fish. Consistently, body length did not differ significantly between paroxetine-treated and control fish (S7 Fig). To assess social behavior, we developed a behavioral assay consisting of a test arena divided into three chambers: a test area, a sibling area, and a non-sibling area (Fig 7A; see Materials and Methods). Following habituation, swimming trajectories of fish in the test area were recorded in the absence (non-sibling trial) or presence (sibling trial) of siblings in the sibling area (Fig 7B). For each frame, we determined whether the fish occupied the sibling-associated region (blue) or the non-sibling-associated region (red) and used these measurements to calculate the social preference index (SPI, −1 < SPI < 1) (Fig 7C; S1-S6 Files; see Materials and Methods) [42]. Total swimming distance did not differ significantly among paroxetine-treated, 5-HT-treated, and control DMSO-treated fish in either non-sibling or sibling trials (Fig 7D). The mean difference in SPI between sibling and non-sibling trials tended to be more negative in both paroxetine- and 5-HT-treated fish than in controls, although neither comparison reached statistical significance (paroxetine, *p* = 0.103; 5-HT, *p* = 0.083; permutation test) (Fig 7E). Behavioral analysis further showed that the proportion of fish exhibiting a positive SPI (sibling trial > non-sibling trial; white) was lower in the paroxetine- and 5-HT-treated groups than in controls, whereas the proportion exhibiting a negative SPI (sibling trial < non-sibling trial; black) was increased (Fig 7F; DMSO, n = 13; parox, n = 7; 5-HT, n = 6). Again, these differences were not statistically significant relative to controls (paroxetine, *p* = 0.195; 5-HT, *p* = 0.096; permutation test). Nevertheless, the results suggest a tendency toward negative social preference in fish exposed to paroxetine or 5-HT during early development. In contrast, we observed a differential effect of paroxetine and 5-HT on total swimming distance. Total swimming distance was significantly reduced during sibling trials in control and paroxetine-treated fish, but not in 5-HT-treated fish (Fig 7G). Consequently, the difference in swimming distance between sibling and non-sibling trials was negative in controls (Fig 7H), a pattern also observed in paroxetine-treated fish. In contrast, this decrease was abolished in 5-HT-treated fish, and the difference was significantly greater than that observed in control and paroxetine-treated fish (control, *p* = 0.0427; parox, *p* = 0.0082; Fig 7G, H). These findings indicate that transient early exposure to paroxetine and 5-HT differentially affects later behavioral responses.

**Fig 7:**
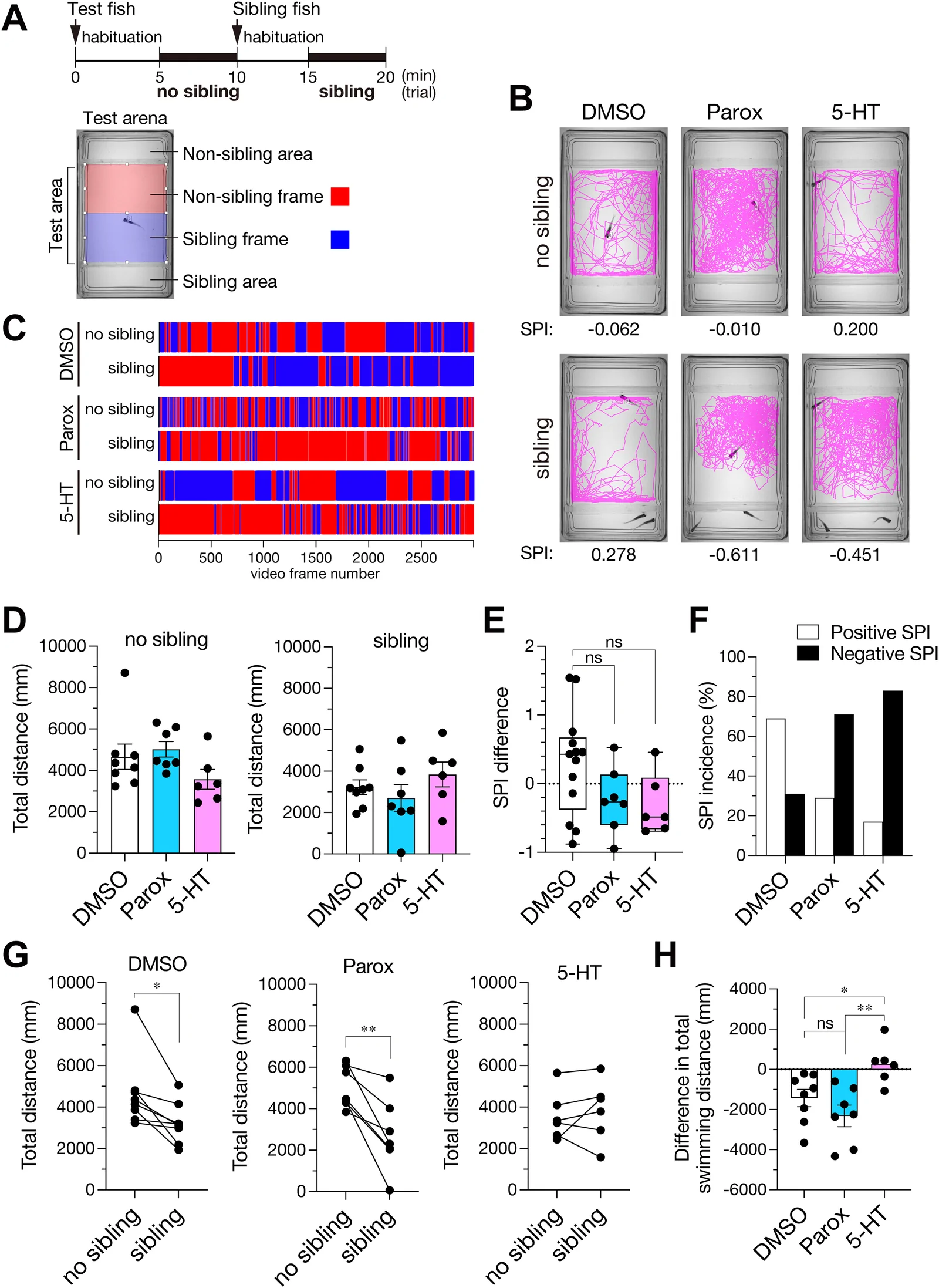
Social behavior of zebrafish exposed to transient early paroxetine or 5-HT. (A) (Top) Schematic diagram of the social behavior assay. (Bottom) Test arena used in this study. Sibling-associated and non-sibling-associated frames are shown in blue and red, respectively. (B) Representative swimming trajectories of fish treated with DMSO (control), paroxetine, or 5-HT during non-sibling (top) and sibling (bottom) trials. The corresponding SPI values are indicated below. (C) A total of 3,000 frames recorded over 5 min for the trials shown in (B), color-coded according to the frame classifications shown in (A). (D) Total swimming distances of DMSO-, paroxetine-, and 5-HT-treated fish during non-sibling (left) and sibling (right) trials. (E) Differences in SPI between sibling and non-sibling trials. Data are presented as mean ± SEM. Permutation test. (F) Incidence of positive SPI (sibling trial > non-sibling trial, white) and negative SPI (sibling trial < non-sibling trial, black) in fish treated with DMSO, paroxetine, or 5-HT (n = 13, 7, and 6, respectively). (G) Changes in total swimming distance between non-sibling and sibling trials in DMSO-, paroxetine-, and 5-HT-treated fish. (H) Differences in total swimming distance between sibling and non-sibling trials. Data are presented as mean ± SEM. *p < 0.05, **p < 0.01; ns, not significant. Brown-Forsythe test with Dunnett’s multiple-comparisons tests. 5-HT, serotonin; ANOVA, analysis of variance; DMSO, dimethyl sulfoxide; GFP, green fluorescent protein; hpf, hours post-fertilization; parox, paroxetine; SEM, standard error of the mean; SPI, social preference index.

## Discussion

In this study, we show that early exposure to paroxetine restricts embryonic growth, resulting in shortened body length and reduced head size in zebrafish. Although we did not functionally validate the effects of paroxetine on zebrafish SERT, we demonstrate strong sequence conservation of SERT between humans and zebrafish. Growth restriction was induced by paroxetine, but not by 5-HT, which is transported into cells through SERT, the target inhibited by paroxetine. Consistent with this observation, paroxetine, but not 5-HT, induced localized apoptosis in the brain and tail tip, suggesting that apoptosis underlies paroxetine-induced embryonic growth restriction. These findings also indicate distinct effects of paroxetine and 5-HT on embryogenesis. SSRIs, including paroxetine, have been reported to induce apoptosis in a variety of malignant cell types, including gliomas, neuroblastomas, leukemias, lymphomas, and colon tumors, as well as in normal cells such as myeloid cells, osteoblasts, and osteoclasts [54–57]. Collectively, these observations suggest a shared mechanism by which SSRIs induce apoptosis in proliferating cells, potentially contributing to their adverse effects on embryonic development. In humans, early-onset FGR is characterized by symmetrical growth restriction with a proportionally small head and body and can result from teratogenic effects [15,16]. Thus, paroxetine-treated zebrafish embryos may serve as a model of human early-onset FGR.

Herein, we show that embryos treated with paroxetine or 5-HT exhibit reduced numbers of post-mitotic neurons in the optic tectum. The effect of paroxetine was reproduced by other SSRIs, suggesting that it results from a shared pharmacological property of these antidepressants, namely serotonin reuptake inhibition. SSRI-treated embryos also exhibited significantly reduced CyclinD1 protein expression. These findings suggest that SSRIs suppress neurogenesis, as Cyclin D1 promotes neurogenesis through a cell cycle-independent mechanism [58]. Despite the reduction in post-mitotic neurons, the number of pH3-positive mitotic cells was selectively increased in the SVZ in paroxetine-treated embryos. We further show that apoptosis is transiently induced and subsequently diminished in the brain of paroxetine-treated embryos. These findings are consistent with a previous report showing that SERT-knockout mice exhibit reduced programmed cell death in the brain [59]. These observations raise the possibility that paroxetine promote the proliferation of neural stem/progenitor cells at the expense of neurogenesis during brain development [24,31,60]. This interpretation is supported by increased SOX2 expression. SOX2 is a transcription factor regulated by the PI3K/AKT signaling pathway that is essential for stem cell induction and maintenance and serves as a marker of proliferating neural stem/progenitor cells in the brain [61,62]. The mechanisms underlying a transition from paroxetine-induced transient apoptosis to increased mitotic cells in the SVZ remain to be clarified. However, our findings are consistent with previous reports showing that SSRIs increase the number of neural stem/progenitor cells in the dentate gyrus of the adult mammalian hippocampus through elevated 5-HT levels, which is thought to be the therapeutic actions of SSRIs in the adult brain [24,52,63]. In addition, our results suggest a biological mechanism that may underlie adverse effects of SSRI exposure on the embryonic brain [11–14] (S8 Fig), although the mechanism by which paroxetine induces apoptosis remains unclear. In rat glioma and human neuroblastoma cell lines, paroxetine and fluoxetine have been reported to cause apoptosis through a rapid increase in p-c-Jun, Cytochrome c release from mitochondria and increased Caspase-3 activity [54]. These data suggest a potential mechanism underlying paroxetine-induced apoptosis in the brain. The similar effects of multiple SSRIs suggest that serotonin uptake through SERT is critical for the survival of neural stem/progenitor cells (S8 Fig).

Although apoptosis induction differs between paroxetine and 5-HT during early development, both treatments cause delayed neurogenesis. Intriguingly, we found that head size was restored at later larval stages in embryos exposed to paroxetine during early development, whereas body length remained reduced, implying brain-preferential regeneration. Consistent with this observation, our data indicate that delayed neurogenesis in the optic tectum reduces both the area and fluorescence intensity of the retinotectal projection in paroxetine-treated embryos, and that these deficits are subsequently restored following paroxetine removal. These findings raise the possibility that neural network reorganization occurs in the larval brain during the regenerative process, as this period coincides with the activity-dependent refinement stage of retinotectal projection development [48,49]. Indeed, we observed extended retinal axon projections in the regenerated brain. This phenotype resembles that reported in SERT-knockout mice, which exhibit impaired segregation of retinal axons in both the superior colliculus and dorsal lateral geniculate nucleus, resulting in disrupted sensory map formation [64,65]. Similar axonal extensions were also observed in the optic tectum treated with 5-HT. These findings suggest that decreased neurogenesis may contribute to the extension of retinal axons, as this phenotype occurs irrespective of whether growth restriction and localized apoptosis are induced. However, the underlying mechanisms by which decreased neurogenesis leads to extended axonal projections remain to be defined. Delayed neurogenesis followed by regeneration was also observed in the telencephalon of paroxetine- and 5-HT-treated embryos using the SAGFF120A gene-trap line. The magnitude of the reduction in fluorescent protein expression differed between SAGFF120A and Tg(*elavl3:Kaede*) embryos at 48 hpf, likely reflecting differences in the neuronal populations labeled by these reporters. In the SAGFF120A line, GFP labels neurons within the telencephalon, including a region corresponding to the mammalian amygdala. These neurons have been shown to play an essential role in active avoidance fear conditioning [29,38]. Therefore, regeneration in the telencephalon suggests that these treatments induce neural circuit reorganization similar to that observed in the optic tectum, potentially affecting neural functions involved in fear conditioning. Notably, individuals with ASD exhibit atypical developmental changes in amygdalar neuron numbers relative to neurotypical individuals [66].

Finally, we investigated the impact of delayed neurogenesis on the development of social behavior. In control fish, SPI differences between sibling and non-sibling trials varied substantially among individuals, and consequently no significant differences were detected between paroxetine- or 5-HT-treated fish and controls. Nevertheless, the incidence of negative SPI (sibling trial < non-sibling trial) was markedly increased in both treatment groups. The absence of significant differences in total swimming distance between paroxetine-treated, 5-HT-treated, and control fish argues against altered locomotor activity as the primary cause of the behavioral changes observed. Although total swimming distance significantly decreased during sibling trials in control and paroxetine-treated fish, a result that could be explained by habituation, habituation alone cannot account for the increased tendency toward negative SPI observed in both paroxetine- and 5-HT-treated fish. Thus, habituation is unlikely to fully explain the behavioral phenotype. In addition, the absence of obvious defects in food intake or feeding behavior, which largely depend on visual function, argues against visual impairment as a major contributing factor [67]. Collectively, these findings suggest that early exposure to paroxetine or 5-HT may negatively influence the development of social behavior. However, these early environmental perturbations alone are unlikely to be sufficient to produce robust behavioral abnormalities, as SPI values did not differ significantly between treatment groups and controls. Nevertheless, transient apoptosis accompanied by delayed neurogenesis may increase susceptibility to neurodevelopmental disorders when combined with other genetic or environmental risk factors. Notably, the effects on total swimming distance differed significantly between paroxetine and 5-HT, suggesting distinct effects on neural circuit function. These findings may provide a biological mechanism linking early developmental risk factors to ASD pathogenesis. However, the interactions between these effects and other risk factors, including sex-specific influences, remain unresolved. Therefore, more detailed analyses will be required to fully understand how paroxetine and 5-HT differentially affect the structure and function of relevant neural circuits. In conclusion, our study provides evidence that transient early exposure to paroxetine and 5-HT influences embryonic brain development and subsequent behavior. Furthermore, this model may serve as a simple screening platform for evaluating risk factors associated with ASD and other neurodevelopmental disorders that affect social behavior.

## Supporting information

S1 Fig Distribution and knockdown of zebrafish SLC6A4a. (A) Immunostaining with an anti-SERT antibody in 30-hpf SLC6A4a-knockdown (KD) embryos generated by injection of slc6a4a antisense MO (AMO) and control embryos injected with 5mis. (B) Quantification of immunofluorescence signals in SLC6A4a-KD embryos. (C) Expression of *slc6a4a* mRNA in SLC6A4a-KD embryos determined by RT-qPCR. (D) Lateral views of 32-hpf embryos injected with antisense slc6a4a-AMO or the control slc6a4a-5mis. White line, head-tail body length. (E, F) Quantification of body length (E) and head area (F) in the injected embryos. (G) Lateral views of 32-hpf Tg(*elavl3:Kaede*) embryos injected with MOs (top) and the corresponding embryos immunostained for active Caspase-3 (bottom). (H) Quantification of active Caspase-3 signal intensity in the brains of the injected embryos. Scale bars: 200 µm (D); 100 µm (A, G). Data are presented as mean ± SEM. *p < 0.05, ***p < 0.001, Mann–Whitney U test.
(TIF)

S2 Fig Development of morphology and the retinotectal projection in wild-type embryos. (A) Lateral views of wild-type embryos at 50 hpf, 72 hpf, 5 dpf, and 7 dpf. White line, head-tail body length. (B, C) Quantification of body length (B) and head area (C) in wild-type embryos. (D) Lateral views of wild-type Tg(*pou4f1-hsp70l:GFP;pou4f3:GAL4;UAS:mCherry*) embryos at 72 hpf, 5 dpf, and 7 dpf. GFP (green) is expressed in tectal neurons and retinal axons. mCherry (red) is expressed in retinal axons. (E, F) Quantification of GFP-positive (E) and mCherry-positive (F) areas in the optic tectum. (G, H) Quantification of GFP (G) and mCherry (H) signal intensity in the optic tectum. Scale bars: 200 µm (A); 100 µm (D). Data are presented as mean ± SEM. *p < 0.05, **p < 0.01, ***p < 0.001, ****p < 0.0001, ns, not significant. Kruskal–Wallis with Dunn’s multiple-comparisons tests.
(TIF)

S3 Fig Effects of early paroxetine on embryogenesis and neurogenesis at 3 dpf. (A) Schematic diagram of treatment with 1 µM paroxetine. (B) Lateral views of 73-hpf embryos treated with paroxetine or the DMSO control. (C, D) Quantification of body length (C) and head area (D) in the treated embryos. (E) Schematic diagram of treatment with 10 µM paroxetine. (F) Lateral views of 72-hpf embryos treated with paroxetine or DMSO control. (G, H) Quantification of body length (G) and head area (H) in the treated embryos. (I) Lateral views of 72-hpf Tg(*pou4f1*-hsp70l:GFP) embryos treated with 10 µM paroxetine or the DMSO control. (J) Quantification of GFP intensity in the optic tectum of the treated embryos. White lines, head-tail body length. Parox, paroxetine. Scale bars: 200 µm (B, F); 100 µm (I). Data are presented as mean ± SEM. *p < 0.05, **p < 0.01, ***p < 0.001, ****p < 0.0001, Mann–Whitney U test.
(TIF)

S4 Fig Effects of early paroxetine and other SSRIs on neurogenesis. (A) Lateral views of a 50-hpf TgBAC(*neurod:EGFP*) embryo. Regions and lines used for intensity quantification are indicated in the bottom image. (B) Intensity profiles along the yellow arrows in (A, bottom) in the optic tectum (green dots) and hindbrain (gray dots). (C) Intensity frequency distributions of the regions (white squares in A, bottom) in the optic tectum (green dots) and hindbrain (gray dots). (D) Schematic diagram of the time course of paroxetine treatment. (E) Lateral views of 50-hpf Tg(*elavl3:Kaede*) embryos treated with 1 or 10 µM paroxetine and the DMSO control. (F-I) Quantification of Kaede-positive area (F, G) and intensity (H, I) in the optic tectum (F, G) and telencephalon (H, I) of embryos treated with 10 µM paroxetine. (J) Lateral views of 50-hpf TgBAC(*neurod:EGFP*) embryos treated with 10 µM sertraline and the DMSO control. (K) Expression of CyclinD1 protein in the optic tectum. Images immunostained for CyclinD1 (red) merged with DAPI nuclear staining (top) and binarized for CyclinD1 signals (bottom). (L) Lateral views of DMSO- and paroxetine-treated embryos immunostained for SOX2 (magenta). (M, N) Quantification of SOX2 protein expression by immunohistochemistry (M) and *sox2* mRNA expression by RT-qPCR (N). Parox, paroxetine. Cb, cerebellum; e, eye; H, hindbrain; OT, optic tectum; T, telencephalon. Filled arrowhead, optic tectum; open arrowhead, telencephalon. Scale bars: 100 µm (A, E, K); 10 µm (J). Data are presented as mean ± SEM. *p < 0.05, **p < 0.01, Mann–Whitney U test.
(TIF)

S5 Fig Growth restriction and decreased neurogenesis are induced by early transient paroxetine at 5 dpf. (A) Schematic diagram of treatment with 10 µM paroxetine and 5-HT. (B) Lateral views of 5-dpf larvae treated with paroxetine, 5-HT, or the control DMSO. White line, head-tail body length. (C, D) Quantification of body length (C) and head area (D) in the treated larvae. (E) Lateral views of 5-dpf Tg(*pou4f1-hsp70l:GFP*) larvae treated with 10 µM paroxetine or the control DMSO. (F, G) Quantification of GFP-positive area (F) and GFP intensity (G) in the optic tectum of the treated larvae. Parox, paroxetine. Scale bars: 200 µm (B); 100 µm (E). Data are presented as mean ± SEM. *p < 0.05, ***p < 0.001, ns, not significant. Kruskal–Wallis with Dunn’s multiple-comparisons tests (C, D), Mann–Whitney U test (F, G).
(TIF)

S6 Fig Decreased neurogenesis in the telencephalon and restoration of head morphology in 7-dpf larvae treated with paroxetine and 5-HT. (A) Schematic diagram of treatment with 10 µM paroxetine and 5-HT. (B) Dorsal views of 48-hpf SAGFF120A;UAS:GFP embryos treated with paroxetine, 5-HT, or the DMSO control. (C) Quantification of mean GFP intensity in the telencephalons of SAGFF120A;UAS:GFP embryos. (D) Schematic diagram of treatment with 1 µM paroxetine and 5-HT. (E) Lateral views of 7-dpf larvae treated with paroxetine, 5-HT, or the DMSO control. White line, head-tail body length. (F, G) Quantification of body length (F) and head area (G) in the treated larvae. Parox, paroxetine. Scale bars: 100 µm (B); 200 µm (E). Data are presented as mean ± SEM. **p < 0.01, ***p < 0.001, ns, not significant. Kruskal–Wallis with Dunn’s multiple-comparisons tests.
(TIF)

S7 Fig Body length of paroxetine-treated and control fish used for social behavioral analyses. (A) Body length of fish at 21 dpf. (B) Body length of fish during 32–36 dpf. Data are presented as mean ± SEM. ns, not significant. Mann–Whitney U test.
(TIF)

S8 Fig Summary of the results obtained in this study. Results obtained following treatment with paroxetine, other SSRIs, and SERT knockdown are shown in light blue. Those obtained following 5-HT treatment are shown in magenta and those observed following both SSRI and 5-HT treatments are shown in black on a pale blue background.
(TIF)

S1 File DMSO-treated fish during a no-sibling trial. (MOV)

S2 File DMSO-treated fish during a sibling trial. (MOV)

S3 File Paroxetine-treated fish during a no-sibling trial. (MOV)

S4 File Paroxetine-treated fish during a sibling trial. (MOV)

S5 File 5-HT-treated fish during a no-sibling trial. (MOV)

S6 File 5-HT-treated fish during a sibling trial. (MOV)
