# Supplementary material for: Paroxetine-induced transient apoptosis with delayed neurogenesis triggers brain-preferential regeneration in developing zebrafish
Source: PLoS One. 2026 Oct 30;21(10):e0345903. doi: 10.1371/journal.pone.0345903 (PMC13722289; doi:10.1371/journal.pone.0345903)
Supplement: Supplementary file 1 [file pone.0345903.s015.docx]

**Response Letter to Reviewers**

PLOS ONE Decision: Revision required 2025/05/19

PONE-D-25-03426

Paroxetine-induced transient apoptosis and delayed neurogenesis act on social behavior in developing zebrafish

Summary of Responses to the Reviewers’ comments:

We greatly appreciate the reviewers’ reasonable and insightful comments on our manuscript. Although it took longer time than 6 months allowed for revision, we revised this manuscript to respond to almost all the comments from the reviewers as much as possible. Briefly,

1) By additional experiments, our results reconfirmed that the effect of paroxetine is reproduced by treatment with other SSRIs including fluoxetine, fluvoxamine and sertraline (Fig 4G and data not shown), suggesting that those effects are attributable to a shared property of SSRIs, serotonin-reuptake inhibition.

2) Immunohistochemistry for Cyclin D1 that plays a role in promoting neurogenesis [71] was performed in paroxetine-treated embryos and showed decreased expression of the proteins in the optic tectum (Fig 4H and S4J Fig), suggesting that neurogenesis is suppressed by paroxetine.

3) Food intake and feeding behavior that mainly depend on visual system [74] appeared normal, and body length as an indicator of growth was unaffected in the treated juvenile fish, suggesting that the possibility of visual impairment is mostly ruled out.

4) The data for social behavioral analysis were statistically reevaluated by a permutation test. This revealed that both the SPI difference and SPI incidence were not significantly different between the control and treated fish, although a tendency for negative SPI is indicated in the treated fish (Fig 7E,F). Therefore, we altered the Title and the description of the results from social behavior in the Abstract, Results and Discussion.

Our responses to each comment are described in “Our answer” sections following the reviewer’s comments below.

**Responses to the Reviewers’ each comment**

**Reviewer #1**: Sato and colleagues describe a series of experiments in larval zebrafish to determine how embryonic exposure to an SSRI (paroxetine) and 5-HT affect the development and behavior of the animals. Overall, the study is thoughtfully designed and executed. My primary concern is that the author's tend to over interpret their findings and to draw conclusions that aren't necessarily supported by the data.

**Major points**
***Comment 1****: The interpretation of figure 3H-J is not convincing. The author's claim that there is a decrease in caspase-3 activity in the optic tectum and dorsal telencephalon following paroxetine washout. However, the decrease in caspase-3 appears to be brain-wide and not specific to these regions. If the author's want to make this claim they would need to quantify all the parts of the brain and show the specificity for these regions. Otherwise, it needs to be discussed appropriately as brain-wide.*

**Our answer**: We completely agree with the reviewer’s comment that the decrease in caspase-3 activity appears to be brain-wide. Actually, we quantified the decrease in the optic tectum and the telencephalon as representative brain regions. Therefore, we added a sentence and words “A brain-wide decrease of Caspase-3-positive signals were detected in the paroxetine-treated embryos.” and “Indeed, as representative brain regions,” (lines 203-205).

***Comment 2****: It is not clear why figure 4 is being interpreted in the context of neurogenesis. All of the cells they have labeled are post-mitotic neurons, they are not looking at the number of proliferating progenitors. Indeed, the prior findings on the increase in caspase-3 (Figure 3A-G) suggest there is considerable apoptosis going on, which would also be expected to result in a decrease in the number of neurons. It seems a more parsimonious explanation based on the data in the paper would be that the reduction in the number of neurons due to paroxetine is due to increased apoptosis, not decreased neurogenesis.*

**Our answer**: We appreciate valuable comments from the reviewer. According to the reviewer’s suggestions, the explanation for Figure 4 was corrected to be a more parsimonious one. The section title was corrected to be “Paroxetine decreases post-mitotic neurons, whilst increasing proliferation of neural progenitor cells in the optic tectum” (lines 227-228). The description of Figure 4D was changed to “suggesting that the number of post-mitotic neurons was significantly decreased due to increased apoptosis by paroxetine (Fig. 3B, D–F) (lines 246-248). We also changed the next sentence to “We then investigated the effect of upregulation of extracellular 5-HT by treating embryos with 5-HT” (lines 253-255). The description of Figure 4E, F was changed to “(Fig. 4E, F), indicating that the effect of paroxetine on the post-mitotic neurons was recapitulated by 5-HT even in the absence of induction of apoptosis (Fig. 3B, D, F).” (lines 257-259). In addition, the words “by suppressing neurogenesis” were removed from the sentence “These results suggest that paroxetine promotes the proliferation of neural progenitor cells in the optic tectum.” (lines 272-273). Furthermore, the words “suppressed neurogenesis were replaced with “decreased (post-mitotic) neurons/neurogenesis” (lines, 275, 306, 497, 469–470, 517, 518–519, 522) or “delayed neurogenesis” (lines, 535, 552) throughout the manuscript.

***Comment 3****: The behavioral data is not convincing with respect to seeing a difference between vehicle and paroxetine or 5-HT treated fish. While figure 7D looks promising, the data in figure 7E is not given no difference between DMSO and paroxetine or 5-HT animals. To be able to draw a conclusion about the behavior, the author's need to demonstrate some sort of statistically significant effect. I would suggest considering a permutation test to determine if the distribution of SPI incidence (Figure 7D) in vehicle treated fish is indeed different from the other groups. In the absence of statistical significance here, the author's cannot draw the conclusion that there is a reduction in social behavior and this needs to be removed from the title and abstract so as not to mislead readers.*

**Our answer**: According to the reviewer’s suggestions, we performed permutation test and found that there were no significant differences in both SPI difference and SPI incidence between the control and treated fish (Fig. 7E, F in the original manuscript). Therefore, we removed all the description for a reduction in social behavior and changed the order of the original Figure 7D and E. Specifically, the title was changed by removing the words “act on social behavior”, and replaced with “Paroxetine-induced transient apoptosis with delayed neurogenesis induces brain remodeling in developing zebrafish” (lines 1-3). The sentence “the paroxetine-treated fish exhibited impaired social behavior, further supporting the correspondence with ASD.” was removed from the abstract and replaced with “social behavior of the paroxetine-treated fish was examined.” (lines 44-45). The phrase “affects typical development of social behavior” was removed from the section title (lines 354-355). The phrase “exhibit atypical social behavior” in the caption of Figure 7 was removed, and the caption was changed to “Social behaviors of early transient paroxetine- and 5-HT-treated zebrafish.” (line 421). We also removed the sentence “Thus, our behavioral analyses demonstrate the negative impact of early exposure to paroxetine and 5-HT” from the discussion (lines 483-484 of the original manuscript) and substituted it with the sentences “our observations raise the possibility of a tendency to negatively impact the development of social behavior by early exposure to paroxetine and 5-HT. However, these early environmental conditions per se are less likely to be sufficient to have a significant effect on social behavior, because the effects on SPIs do not show significant differences.” (lines 548-552).

***Comment 4****: The author's mention that they are observing changes in the 'dorsal telencephalon' several times throughout the manuscript. However, from the images, it is not clear that they can actually differentiate the dorsal from the ventral telencephalon. This needs to be changed throughout the manuscript to refer just to the telencephalon.*

**Our answer**: According to the reviewer’s helpful comments, the word “dorsal telencephalon” was corrected to refer just to the “telencephalon” throughout the manuscript.

***Comment 5****: Related to the above point, the author's make use of the SAGFF120A line to label the telencephalon in zebrafish. There are several issues with their claims based on this line. Firstly, the paper they cite for this labelling the medial dorsal telencephalon (Dm; Lal et al, 2018) looked at expression in this enhancer trap line in adults. And while the expression is specific to the Dm in adults, it's not clear if this is also true in larval animals. Indeed, the larval dorsal telencephalon does not contain clear cytoarchitectonic boundaries to differentiate the Dm (putative basolateral amygdala homolog) from other regions like the Dc, or Dl. Thus, it is not appropriate to draw the conclusion that the zebrafish homolog to the amygdala is being labelled.*

**Our answer**: We appreciate the reviewer’s insightful comments. According to the comments, we corrected all the description of the telencephalon labeled in the SAGFF120A to refer to the phrases “the telencephalon that includes a region corresponding to the mammalian amygdala” throughout the manuscript (lines 38-39 in the abstract, 357 in the results, 527-528 in the Discussion).

***Comment 6****: Secondly, the author's do not actually see a decrease in GFP signal in the SAGFF120A line. On line 340-342 the author's state that "Fluorescence of the GFP-expressing areas was significantly decreased 341 in the dorsal telencephalons of paroxetine- and 5-HT-treated embryos compared to the 342 control at 48 hpf (5-HT, p = 0.00161, Fig. 6A, B)" However, the difference in figure 6A between vehicle and paroxetine fish is not different. This actually argues against the dorsal telencephalon/Dm being specifically affected by paroxetine as they saw in figure 4. The paper needs to change all the language suggesting that there was a paroxetine induced change in the dorsal telencephalon/amygdala homolog.*
**Our answer**: Thanks to the reviewer’s precise comments, we noticed that our explanation for Figure 6A, B was inadequate (lines 343-345 of the original manuscript). Correctly, fluorescence of GFP-expressing areas was not significantly decreased in the telencephalon of the paroxetine-treated SAGFF120A embryos at 48 hpf (the original Fig 6A, B) but significantly decreased at 76 hpf (the original S6B, D Fig). Once again, by an additional experiment, we confirmed that the GFP expression was slightly but not significantly decreased in the telencephalon of paroxetine-treated SAGFF120A;UAS:GFP embryos compared to the control at 48 hpf (see below).

These results suggest that a decrease in GFP expression was not sufficient to show a significant decrease at 48 hpf, but shows a significance at 76 hpf. To circumvent a misunderstanding of our results, we exchanged the results at 48 hpf that were originally shown in Figure 6A, B to those at 76 hpf in S6A­–E Fig. In concordance with this correction, the description in the Results was changed to “Fluorescence of the GFP-expressing areas was significantly decreased in the telencephalons of paroxetine- and 5-HT-treated embryos compared to the control at 76 hpf (parox, *p* = 0.0279; 5-HT, *p* = 0.0250, Fig 6B–E) as observed in the optic tecta, although the decrease was significant for 5-HT but not for paroxetine at 48 hpf (parox, *p* = 0.3344; 5-HT, *p* =0.0006, S6 Fig)” (lines 362-366). These results indicate a difference in the extent of a decrease in the expression of fluorescent proteins in the telencephalon at 48 hpf, since these transgenic lines label different populations of neurons in the telencephalon under the control of different regulatory elements, that is SAGFF120A-trapped gene and pan-neuronal *elavl3* (*huC*) promotor. Thus, we don’t think that the results in the SAGFF120A shown in the original Figure 6A, B argue against those in Tg(*elav3l:Kaede*) in S4 Fig E, I. However, for clarity, we added the above-mentioned view of the difference in the telencephalon in the Discussion as follows: The extent of the decrease in the fluorescent protein expression is different in the telencephalon of SAGFF120A from that of Tg(*elavl3:Kaede*) at 48 hpf. This is likely due to the difference in the population of neurons labeled by the fluorescent proteins (lines 524-528).

**Minor points**
**Comment 7**: Where possible, real measurements instead of arbitrary units should be used (e.g., in the measurement of the body length and head area).
**Our answer**: According to the reviewer’s useful comment, the graphs showing the body length and head area (Fig. 2D, E, H and I) were replaced with those using real measurements (µm and µm^2^, respectively) instead of arbitrary units (a.u.).

***Comment 8****: How does the amount of paroxetine used in this study compare to what the fetus in humans is exposed to?*
**Our answer**: The concentration of paroxetine that the human fetus is expose to is roughly estimated by the ratio of placental passage of antidepressant medications (Ref. 9: Hendrick, Am J. Psychiatry, 2003). The mean rations of umbilical cord to maternal serum concentrations of antidepressants ranged from 0.29 to 0.89. Paroxetine pharmacokinetics is reported that the maximal blood concentration (C_max_) and the maximal time point (T_max_) are 1.93 ± 1.38 ng/mL and 4.61 ± 1.04 h, respectively, in adult healthy humans at a single dose of 10 mg paroxetine orally (Paxil Tablet, GSK; Irie, Jpn Pharmacol. Ther. 28(S1): 47-68 (2000)). Our treatment with 1–20 µM paroxetine by bath application in E3 medium corresponds to 0.3748 and 7.4966 µg/mL *in vitro*, and thus approximately 200–3900 times higher than the C_max_ in the blood. We added this estimation in the methods (lines 610-615).

***Comment 9****: In Figure 3F I would recommend writing "paroxetine + 5-HT" instead of "paroxetine/5-HT" as the latter suggests you are dividing paroxetine by 5-HT in some way.*

**Our answer**: According to the reviewer’s useful advice, we corrected the Figure 3F, G by indicating *"paroxetine+5-HT" instead of "paroxetine/5-HT"*.

***Comment 10****: It would be helpful if the bold rectangle in figure 1C drawing was oriented the same way as the microscopy images.*

**Our answer**: According to the reviewer’s helpful comment, we changed the orientation of Figure 1C to conform to the microscopy images in the right.

***Comment 11****: Line 442 is awkward and needs to be fixed.*

**Our answer**: According to the reviewer’s exact comment, the awkward sentence was replaced by the sentences “The effect of SSRI is thought to represent the underlying mechanism of the therapeutic effect on the maternal brain. On the other hand, our results suggest a biological mechanism that underlie an adverse effect on the embryonic brain which may be caused by SSRI ingestion during pregnancy.” (lines 486–489).

***Comment 12****: Line 451, we do not to 'proofs' in science, only in math. Please use a different word other than 'prove', for example 'we provide support for' or 'our data supports'.*

**Our answer**: According to the reviewer’s accurate comment, the word “prove” was changed to “our data support” (line 506).

***Comment 13****: Lines 467-471, these parts of the discussion need to be re-written since there is no evidence for changes in the Dm region of the zebrafish in this paper due to paroxetine.*

**Our answer**: In response to the Comment 6 from the reviewer, we added the description mentioned above in the discussion (Please see Our answer to the Comment 6).

***Comment 14****: Lines 484-486: why are the author's interpreting a chance in locomotor activity as a change in social behavior? Because of the experimental design, this decrease in overall activity is likely due to habituation to the environment. Because the author's did not counterbalance exposure to the sibling zebrafish (eg some animals got no sibling first, and others got sibling first), effects of change in locomotion over time cannot be ruled out. This interpretation needs to altered along with the title and abstract of the paper.*
**Our answer**: In the social behavioral analysis, we took time to habituate a test fish to the test area and the siblings for each 5 min and then recorded the swimming behavior for 5 min as mentioned in the method (lines 679–683). To clarify this procedure for the social behavioral test, we added a schematic diagram in the Figure 7A top (line 422) and the words “following habituation” in the results (line 379).

We actually find that there are no significant differences in the total swimming distances between paroxetine-, 5-HT-treated and the control fishes both in non-sibling and sibling trials). This was exhibited in Figure 7D and explained in the results (lines 383–386). These results would exclude the possibility that the changes in social behavior are due to changes in locomotor activity. This interpretation was described as “No significant differences in the total swimming distances between paroxetine-, 5-HT-treated and the control fishes would exclude the possibility that the changes in social behavior are due to those in locomotor activity.” in the discussion (lines 540–542). Meanwhile, total swimming distances are significantly decreased in sibling trials of the control DMSO and Paroxetine-treated fish, but not in 5-HT-treated fish, which was exhibited in Figure 7G and explained in the results (lines 398–400). These observations may be due to habituation. If so, those cannot explain a similar tendency for the increased negative SPI between Paroxetine- and 5-HT-treated fish. Thus, our observations will exclude the possibility of habituation. These interpretations were described in the discussion (lines 542–545). Together with above-mentioned correctios, the words “involving social behaviors” (line 489 in the original manuscript) were removed and replaced with “functions” (lines 556).

***Comment 15****: Line 573 'until' should be 'unless'*
**Our answer**: The word “until” was replaced with “unless” (line 654).

***Comment 16****:* Line 593 the word 'case' should be replaced with 'tank' or 'arena' here and in other parts of the manuscript referring to the testing apparatus.

**Our answer**: According to the reviewer’s helpful suggestion, the word “case” was replaced with “arena” throughout the manuscript.

**Reviewer #2**: There is a suggested association between SSRI use during pregnancy and an increased risk of ASD in children, and this study uniquely and interestingly explores that association using zebrafish. The main findings indicate that paroxetine exposure induces transient apoptosis and delays neurogenesis, leading to growth restriction and impaired social behavior. Although neurogenesis eventually recovered, suggesting neural circuit remodeling—such as the extension of the retina-tectum projection—abnormal social behaviors were ultimately observed, indicating a potential association with ASD.

**Major Points**
***Comment 1****:* *Overgeneralization regarding direct relevance to humans
The authors strongly suggest a direct involvement of SSRIs in ASD risk based on the zebrafish model results; however, the presented data are insufficient to establish causality. The observed behavioral abnormalities (reduced SPI) cannot be conclusively interpreted as ASD-like behavior. Moreover, the possibility of visual impairment should be ruled out.*

**Our answer**:

We agree that the observed behavioral abnormalities (reduced SPI) cannot be conclusively interpreted as ASD-like behavior. Meanwhile, we believe that our results imply a biological mechanism underlying early exposure to paroxetine, a risk factor associated with the pathogenesis of ASD. To exclude misinterpretations, we corrected several descriptions in our manuscript as follows. The sentence in the abstract “the paroxetine-treated fish exhibited impaired social behavior, further supporting the correspondence with ASD.” (lines 41-42 of the original manuscript) was removed and replaced with “social behavior of the paroxetine-treated fish was examined.” (line 45). The descriptions in the Abstract “Our findings offer new insights into the early neurodevelopmental etiology of ASD.” (lines 42-43 in the original manuscript) were changed to “Our findings imply a biological mechanism underlying early exposure to paroxetine, a risk factor associated with the pathogenesis of ASD.” (lines 45-47). Also, the description in the Discussion “These findings offer new insights into the neurodevelopmental mechanisms underlying the pathogenesis of ASD.” (lines 489-491 of the original manuscript) was replaced with “These findings may offer a biological mechanism underlying early risk factors associated with the pathogenesis of ASD.” (lines 556-557).

In zebrafish, pray capture is known to be dependent on the visual system (Gahtan, J. Neurosci., 2005). We observed no obvious differences in food intake (paramecia and brine shrimp) and feeding behavior in the paroxetine- and 5-HT-treated fish. Indeed, the body length showed no significant differences between paroxetine-treated and the control fish used for behavioral analysis (see below).

Therefore, we think that the possibility of visual impairment can mostly be ruled out. These observations and the interpretation were described as “This is because no obvious differences in food intake and feeding behavior were observed between paroxetine-, 5-HT-treated and the control fish. Indeed, the body length of paroxetine-treated fish was not significantly different from that of the control fish (data not shown).” in the Results (lines 372-375) and “In addition, our observations of no obvious defects of the treated fish in food intake and feeding behavior which depend on the visual system mostly rule out the possibility of visual impairment.” in the Discussion (lines 545-547), respectively.

***Comment 2****:* *Insufficient distinction between the mechanisms of paroxetine and 5-HT
While both paroxetine and 5-HT exhibit similar effects on behavior and neurodevelopment, they also produce distinct outcomes, such as apoptosis induction. However, the mechanisms underlying these differences are not sufficiently explored or discussed. There is also a lack of in-depth investigation into how paroxetine directly induces apoptosis. Additionally, no analysis was performed on the signaling pathways responsible for delayed neurogenesis or on the expression of cell cycle regulators (e.g., Cyclin D1). Although 5-HT administration was tested, comparisons with other SSRIs (e.g., fluoxetine) are lacking, making it difficult to determine whether these effects are specific to serotonin re-uptake inhibition.*

**Our answer**:
We appreciate insightful comments from the reviewer. According to the comments, we performed additional experiments to confirm the effects of other SSRIs and showed the results in Figure 4G and 4H. Consistent with these corrections, we added the description as follows: “Treatment with other SSRIs, fluoxetine, fluvoxamine and sertraline also showed decreased post-mitotic neurons in TgBAC(*neurod1:EGFP*) similar to that with paroxetine, indicating that the effects on post-mitotic neurons were shared with other SSRIs (Fig. 4G and data not shown).” in the Results (lines 248-251), and “The effect of paroxetine is reproduced by the treatment with other SSRIs, suggesting that those effects are attributable to a shared property of antidepressants, serotonin re-uptake inhibition.” in Discussion (lines 470-472).

Apoptosis is induced through intrinsic and extrinsic signaling pathways, and both of which activate an effector caspase, Caspase-3 (Newton, Cell, 2024). At present, we do not know which pathway is activated to induce apoptosis by serotonin re-uptake inhibition through SERT. However, our results suggest that incorporation of serotonin is criticall for survival of neural stem/projenitor cells. The signaling pathway for apoptosis induction by SSRIs is under investigation as the next project, because apoptosis induction is a common feature of the teratogenic effects of SSRI (this study) and thalidomide (Ando, iScience, 2019), in addition to immune responses by infection and inflammation, all of which are risk factors for ASD during pregnancy (Al-Haddad, Am. J. Obstet. Gynecol. 2019). Therefore, we discussed the distinct outcomes in apoptosis induction as follows: “Although there is a difference in apoptosis induction at early stages between paroxetine and 5-HT, both of which cause delayed neurogenesis. This implies that the delayed neurogenesis is a leading cause of the later effects on the retinotectal projection and social behavior, because both the treatments show a similar tendency for significant extension and negative SPI, respectively.” (lines 500-504), and “However, at present, it remains unclear how paroxetine induces apoptosis. Our results showing a similar effect of SSRIs on the post-mitotic neurons, that is decreased neurogenesis, suggest that incorporation of serotonin through SERT will be critical for survival of neural stem/progenitor cells leading to generation of neurons.” (lines 495-499).

Although the signaling pathways responsible for delayed neurogenesis have not yet been elucidated, we showed that the expression of SOX2 proteins was significantly increased in the paroxetine-treated brain, as indicated by S4K–M Fig. The result was described as “Expression of SOX2, a high-mobility group (HMG)-box transcription factor to suppress cell differentiation and promote self-renewal in proliferating neural stem/progenitor cells, was enhanced in paroxetine-treated embryonic brains, with a concomitant decrease in Kaede- expressing neurons at 48 hpf,” (lines 267-270) in the Results and in the Discussion as “This is supported by increased expression of SOX2, a transcription factor regulated by the PI3K/AKT signaling pathway that is not only inevitable for the induction and maintenance of stem cells, but also a marker of proliferating neural stem/progenitor cells in the brain.” (lines 480-483).

In addition, we performed immunohistochemistry using anti-CyclinD1 antibody and showed a significant decrease in the expression of CyclinD1 proteins in the optic tectum of the SSRI-treated embryos, as exhibited in Fig. 4H and S4J Fig, and described in the Results and the Discussion as follows: “Immunohistochemistry for CyclinD1 showed a significant decrease in the protein expression in the optic tectum of the SSRI-treated embryos (Fig. 4H and S4 Fig).” (lines 252-253) and “The SSRI-treated embryos also show a significant decrease in CyclinD1 protein expression. These results imply that neurogenesis is suppressed by SSRIs, because Cyclin D1 play a role in promoting neurogenesis as a cell cycle-independent function [71].” (lines 472-475), respectively.

Moreover, we also performed treatment and comparisons with other SSRIs (fluoxetine, fluvoxamine, sertraline). Our results showed similar effects as paroxetine on post-mitotic neurons and Cyclin D1 expression, suggesting that those effects are attributable to a shared property of antidepressants, serotonin re-uptake inhibition, as described above.

***Comment 3****: Validation of the social behavior assay is lacking
The social behavior test system used (preference for sibling vs. non-sibling fish) is original and innovative but differs from standardized ASD-like behavioral indices. Including data from conventional behavioral assays would help validate this novel approach.
Moreover, only short-term behavioral changes during the juvenile period (35–37 dpf) were analyzed; persistent behavioral changes in adult fish were not assessed. Since ASD is a lifelong disorder and not merely a developmental delay, it is important to evaluate whether prenatal paroxetine exposure in zebrafish can induce ASD-like symptoms later in life.*

**Our answer**:
We appreciate the reviewer’s reasonable comments. Currently, we are attempting to refine our social behavioral test to be more stable and sensitive, discriminating sensory cues. Unfortunately, we do not have sufficient research resources to reliably evaluate our results using other behavioral assays at present. Thus, validation of our results using conventional behavioral assays is the next project to do for us.

We completely agree with the reviewer’s comment that ASD is a lifelong disorder and not merely a developmental delay. However, in this study, we focus on the effects of the early exposure to paroxetine on neurodevelopment and social behavior at juvenile stages, since our goal is to identify potential biomarkers for the early diagnosis of the risk for ASD. Although we understand the importance of evaluating whether prenatal paroxetine exposure can induce ASD-like symptoms in adult fish, such kinds of experiments are out of focus for this study.
