# Supplementary material for: Paroxetine-induced transient apoptosis with delayed neurogenesis triggers brain-preferential regeneration in developing zebrafish
Source: PLoS One. 2026 Oct 30;21(10):e0345903. doi: 10.1371/journal.pone.0345903 (PMC13722289; doi:10.1371/journal.pone.0345903)
Supplement: Supplementary file 2 [file pone.0345903.s016.docx]

**Response Letter to Reviewers**

PONE-D-26-05926
Paroxetine-induced transient apoptosis with delayed neurogenesis triggers brain-preferential regeneration in developing zebrafish

Tomomi Sato

PLOS One

Summary of Responses to the Reviewers’ comments:

**Responses to the Reviewers’ each comment**

**Reviewer #1**: This manuscript examines the impact of early embryonic exposure to the SSRI paroxetine on neurodevelopment using a zebrafish model. The authors report that paroxetine triggers early apoptosis, alters neurogenesis, and leads to later changes in brain morphology and retinal projections, which are interpreted as brain-preferential remodeling. The study also looks at how these effects compare to 5-HT exposure to explore potential underlying mechanisms. The study addresses an important question regarding the direct effects of SSRI exposure during development and offers a dataset that integrates imaging, genetic reporters, and behavioral assays. The work is generally well-executed and suitable for PLOS ONE, but some parts of how the data is interpreted and how clear it is need to be changed.

**Major comments**
***Comment 1****: The manuscript does not clearly distinguish whether the observed phenotypes arise from decreased intracellular serotonin (due to SERT inhibition) or increased extracellular serotonin signaling. The differential effects of paroxetine and 5-HT (e.g., apoptosis observed only with paroxetine, but neurogenesis defects seen with both) are not sufficiently explained. A clearer and more cautious interpretation of these mechanistic differences is needed.*

**Our answer**: Thank you for the reviewer’s critical comments. We interpret our results to mean that the apoptosis only induced by paroxetine is due to decreased intracellular serotonin by SERT inhibition, whereas the decreased neurogenesis observed both in paroxetine and 5-HT is primarily caused by the apoptosis for paroxetine as described in the Results “suggesting that increased apoptosis induced by paroxetine reduced the number of post-mitotic neurons (Fig 3B, D-F)” (lines 382–383). However, the decreased neurogenesis is also observed in 5-HT without apoptosis induction, implying that it is potentially caused by neural stem/progenitor cell expansion due to increased extracellular serotonin for 5-HT. Consistently, we detected an increase in the number of pH3-positive mitotic cells in the SVZ (Fig. 4I, J) and SOX2 expression (S4K, L Fig). Therefore, according to the reviewer’s valuable comments, we added cautious interpretation of the differential effects of paroxetine and 5-HT in the Results as follows: “This observation implied that reduced neurogenesis can potentially result from expansion of neural stem/progenitor cells.” (lines 393–394) and “these results suggest that paroxetine secondarily promotes the proliferation of neural progenitor cells following apoptosis and consequently impairs neurogenesis in the optic tectum.” (lines 407–409). Furthermore, to define our interpretation more clearly, we added a sentence in the Discussion as follows: “The mechanisms underlying a transition from paroxetine-induced transient apoptosis to increased mitotic cells in the SVZ remain to be clarified.” (lines 609–611). We also removed phrases from the Discussion as follows: “This may provide insight into a biological basis for the neurodevelopmental disorders associated with early-onset FGR.” (lines 745–746, in a marked-up copy), and “and increased 5-HT” (line 689, in a marked-up copy).

***Comment 2****: Figure 1 presents sequence and structural conservation of SERT between human and zebrafish. However, no functional validation of paroxetine on zebrafish SERT is provided. The authors should acknowledge this limitation.*

**Our answer**: According to the reviewer’s helpful comment, we added the description that “Although we did not functionally validate the effects of paroxetine on zebrafish SERT, we demonstrate strong sequence conservation of SERT between humans and zebrafish.” in Discussion (lines 438–440).

***Comment 3****: The increase in proliferating (pH3-positive) cells alongside reduced neuronal differentiation suggests altered progenitor dynamics, but the underlying mechanism is unclear. It is not evident whether this reflects accumulation of progenitor populations or impaired neuronal differentiation.*

**Our answer**: We showed that paroxetine caused an increase in proliferating (pH3-positive) cells in the SVZ (Fig 4I, L), concomitant with reduced neuronal differentiation (Fig 4E, F) and enhanced expression of SOX2 that promotes self-renewal of neural progenitor cells (S4K­­, L Fig). These results suggest that accumulation of progenitor populations consequently leads to impaired neuronal differentiation. In addition, those results explain the reason why GFP-positive neurons are subsequently restored at later stages in the optic tectum and the telencephalon (Fig 5F, ­H and Fig 6F, H) . To make the underlying mechanism clear, we deleted the phrase “suppress cell differentiation and” (line 443) and changed the description to “these results suggest that paroxetine secondarily promotes the proliferation of neural progenitor cells following apoptosis and consequently impairs neurogenesis in the optic tectum.” (lines 268–270).

***Comment 4****: The interpretation of increased retinal projection area as evidence of “brain remodeling” appears overstated and should be toned down. The analysis of retinotectal projections is primarily based on fluorescence area and intensity, which does not distinguish between functional circuit restoration and aberrant axonal growth. The observed expansion of retinal projections at later stages could reflect miswiring or impaired pruning rather than true remodeling. I suggest the authors to revise the language and present these findings more cautiously as alterations in projection patterns rather than definitive remodeling.*

**Our answer**: Thank you for the reviewer’s reasonable comments. According to the comments, we replaced the word “brain remodeling” in the title with “brain-preferential regeneration” (lines 2–3) and the phrase “suggesting brain-preferential remodeling” in the Abstract with “suggesting brain-preferential regeneration” (line 34). We changed the words “remodeling” to “regeneration” in the Abstract (line 32), the Results (lines 303, 460, 461, 503), the Fig 5 and 6 captions (line 470, 542, respectively) and the Discussion (lines 628, 642, 649). In addition, the word “remodeling” in the Discussion was replaced with “reorganization” (lines 632 and 650) and the words “remodeling period” in the Discussion with “this period” (line 633).

***Comment 5****: The observation that 5-HT phenocopies certain aspects of the paroxetine-induced phenotype (e.g., altered neurogenesis and projection changes) is not mechanistically explained. The authors should clarify whether 5-HT is being used as a control, or comparator, and adjust their interpretation accordingly.*

**Our answer**: We appreciate the reviewer’s insightful comments. We used 5-HT as a comparator in order to segregate the direct from the indirect effects of paroxetine, that is, the SERT-inhibiting effect from the resulting effect of increased extracellular 5-HT levels. Because, those effects are supposed to be the underlying mechanisms for the therapeutic effect of SSRIs including paroxetine as antidepressants. This purpose was added in the Introduction as follows: “In this study, we used 5-HT as a comparator of paroxetine in order to segregate the direct from the indirect effects of paroxetine, that is, the SERT-inhibiting effect from the resulting increased extracellular 5-HT levels.” (lines 86–89). By comparing paroxetine with 5-HT, we investigated which and how effects of paroxetine act on brain development. Unexpectedly, we found that both the treatment with paroxetine and 5-HT had similar effects on neurogenesis and projection. Our interpretation of those results has been described in the Discussion as follows: “These findings suggest that decreased neurogenesis may contribute to the extension of retinal axons, as this phenotype occurs irrespective of whether growth restriction and localized apoptosis are induced.” (lines 639–641). Because their molecular mechanisms remain unknown, we added the description as follows: “However, the underlying mechanisms by which decreased neurogenesis leads to extended axonal projections remain to be defined.” (lines 641–642).

**Minor comments**

***Comment 1****: The use of terms such as “restoration” should be reconsidered and replaced with more neutral descriptions.*

**Our answer**: According to the reviewer’s comment, we replaced the word “restoration” with more neutral descriptions such as “regrowth” or “regeneration” depending on the context.

***Comment 2****: What links SERT inhibition to apoptosis? A brief discussion on this would be informative.*

**Our answer**: According to the reviewer’s helpful comment, we added a brief discussion on the potential mechanisms of paroxetine-induced apoptosis as follows: In rat glioma and human neuroblastoma cell lines, paroxetine and fluoxetine have been reported to cause apoptosis through a rapid increase in p-c-Jun, Cytochrome c release from mitochondria and increased Caspase-3 activity [51]. These data suggest a potential mechanism underlying paroxetine-induced apoptosis in the brain. (lines 617–621).

***Comment 3****: Given the known sex- specific differences in neurodevelopmental disorders such as ASD, it would be useful for the authors to briefly discuss whether sex-specific effects could influence their findings. While this may not be feasible to assess at early developmental stages in zebrafish, acknowledging this as a limitation would strengthen the manuscript.*

**Our answer**: We appreciate useful comments from the reviewer. Although we understand the importance of sex-specific differences in neurodevelopmental disorders such as ASD, such sex-specific effects are not considered in this study because of the difficulty of identifying those effects at early zebrafish embryos. Therefore, we acknowledged this as a limitation in the Discussion as follows: “However, the interactions between these effects and other risk factors, including sex-specific influences, remain unresolved.” (lines 679-680).

***Comment 4****: The manuscript would benefit from general language editing to correct grammatical errors and improve clarity (e.g., typographical errors such as “occurrs” (line no.165) and inconsistent terminology (e.g., “parox” vs “paroxetine)).*

**Our answer**: According to the reviewer’s comment, we corrected for the typographical errors such as “occurrs” with “occurs” and for the inconsistent terminology including “parox” with consistently use the term “paroxetine” in the main text. We also utilized English proofreading by ENAGO to correct grammatical errors in our revised manuscript. Our corrections are indicated in light blue, those in response to the reviewer’s comments in light green, and those according to ENAGO in red in a marked-up copy of the revised manuscript.

**Reviewer #2**:

The manuscript “Paroxetine-induced transient apoptosis with delayed neurogenesis induces brain remodeling in developing zebrafish" by Sato and colleagues presents an interesting investigation by demonstrating that early exposure of zebrafish embryos to paroxetine retards neurogenesis in the optic tectum and the telencephalon.

The work is technically solid, the experimental execution sound, and the results show that paroxetine-treated embryos exhibit impaired growth, with small heads and short body lengths resulting from transient apoptosis in the animal model.

The article is very impressive, contributing to a deeper understanding of the biological mechanisms underlying early exposure to paroxetine, which, as the authors argue, constitutes a risk factor implicated in the pathogenesis of autism spectrum disorder (ASD).

The topic is timely and relevant to neurodevelopmental disorders and the use of zebrafish to study these conditions. The manuscript introduces fundamentally new mechanisms that would significantly advance current understanding.

The quantity of results presented in the main article and in the supplementary material is commendable. The discussion, in turn, clearly presents the experimental findings and thoughtfully situates them within the context of existing literature. This approach effectively demonstrates consistency with prior studies and reinforces the reliability of the results. Besides, careful comparison with previous work contributes to a coherent narrative and supports the manuscript's scientific relevance, thereby enhancing its overall clarity and scholarly value.

Lastly, starting on pg. 90, the authors included their responses to the Reviewers’, clearly demonstrating that all suggestions in the previous round have been carefully addressed. They explain that it took longer than six months to revise and address reviewers' comments. This thorough and transparent response reflects the authors’ commitment to improving the manuscript and strengthens the work's overall quality and rigor.

In the opinion of this referee, the only remaining question raised by referees is the overgeneralization of results to humans.

Therefore, I recommend that the authors re-revise their work to interpret their findings and draw conclusions that are supported by their data.

**Our answer**:

Thank you for the reviewer’s critical comments. Because we had obtained new insights from this study into another project exploring early biomarkers of maternal depression, we described much speculation about generalization to humans. According to the reviewer’s comments, we deleted most of the description of the overgeneralization of results to humans from the Discussion, such as the description of fluvoxamine in COVID-19 deterioration, thalidomide teratogenic effects in offspring, and maternal inflammation through DAMPs. We also deleted any speculation from schematic in S8 Fig. However, we left the description of FGR and associations with ASD in humans and translocated those descriptions to the last part of the paragraph in the Discussion. Next, we simplified the Discussion by eliminating redundancy and rearranged it into four paragraphs as follows:

1) Paroxetine-induced FGR

2) Paroxetine-induced apoptosis

3) Proxetine/5-HT-induced delayed neurogenesis

4) Paroxetine/5-HT-triggered impact on social behavior
