# Supplementary material for: Paroxetine-induced transient apoptosis with delayed neurogenesis triggers brain-preferential regeneration in developing zebrafish
Source: PLoS One. 2026 Oct 30;21(10):e0345903. doi: 10.1371/journal.pone.0345903 (PMC13722289; doi:10.1371/journal.pone.0345903)
Supplement: Peer review history

# Author comment: Author response to Decision Letter 0

**DOI:** 10.1371/journal.pone.0345903.r001

11 Mar 2026

Attachment Submitted filename: Response letter_202600202.docx

# Peer review: Decision Letter 0

**DOI:** 10.1371/journal.pone.0345903.r002

**Authors:** Suresh Yenugu

24 Apr 2026

Dear Dr. Sato,

While the study appears to be well designed and presents important research findings, it is advised to limit the interpretations based on the observed results. Also, provide mechanistic insights based on the results observed.

Please submit your revised manuscript by Jun 08 2026 11:59PM. If you will need more time than this to complete your revisions, please reply to this message or contact the journal office at plosone@plos.org. When you're ready to submit your revision, log on to https://www.editorialmanager.com/pone/ and select the 'Submissions Needing Revision' folder to locate your manuscript file.

- A letter that responds to each point raised by the academic editor and reviewer(s). You should upload this letter as a separate file labeled 'Response to Reviewers'.
- A marked-up copy of your manuscript that highlights changes made to the original version. You should upload this as a separate file labeled 'Revised Manuscript with Track Changes'.
- An unmarked version of your revised paper without tracked changes. You should upload this as a separate file labeled 'Manuscript'.

If you would like to make changes to your financial disclosure, please include your updated statement in your cover letter. Guidelines for resubmitting your figure files are available below the reviewer comments at the end of this letter.

If applicable, we recommend that you deposit your laboratory protocols in protocols.io to enhance the reproducibility of your results. Protocols.io assigns your protocol its own identifier (DOI) so that it can be cited independently in the future. For instructions see: https://journals.plos.org/plosone/s/submission-guidelines#loc-laboratory-protocols. Additionally, PLOS ONE offers an option for publishing peer-reviewed Lab Protocol articles, which describe protocols hosted on protocols.io. Read more information on sharing protocols at https://plos.org/protocols?utm_medium=editorial-email&utm_source=authorletters&utm_campaign=protocols.

As the corresponding author, your ORCID iD is verified in the submission system and will appear in the published article. PLOS supports the use of ORCID, and we encourage all coauthors to register for an ORCID iD and use it as well. Please encourage your coauthors to verify their ORCID iD within the submission system before final acceptance, as unverified ORCID iDs will not appear in the published article. *Only*  the individual author can complete the verification step; PLOS staff *cannot*  verify ORCID iDs on behalf of authors.

We look forward to receiving your revised manuscript.

Kind regards,

Suresh Yenugu

Academic Editor

PLOS One

Journal requirements:

When submitting your revision, we need you to address these additional requirements.

1. Please ensure that your manuscript meets PLOS ONE's style requirements, including those for file naming. The PLOS ONE style templates can be found at

https://journals.plos.org/plosone/s/file?id=wjVg/PLOSOne_formatting_sample_main_body.pdf and

https://journals.plos.org/plosone/s/file?id=ba62/PLOSOne_formatting_sample_title_authors_affiliations.pdf

2. When completing the data availability statement of the submission form, you indicated that you will make your data available on acceptance. We strongly recommend all authors decide on a data sharing plan before acceptance, as the process can be lengthy and hold up publication timelines. Please note that, though access restrictions are acceptable now, your entire data will need to be made freely accessible if your manuscript is accepted for publication. This policy applies to all data except where public deposition would breach compliance with the protocol approved by your research ethics board. If you are unable to adhere to our open data policy, please kindly revise your statement to explain your reasoning and we will seek the editor's input on an exemption. Please be assured that, once you have provided your new statement, the assessment of your exemption will not hold up the peer review process.

3. We note that you have included the phrase “data not shown” in your manuscript. Unfortunately, this does not meet our data sharing requirements. PLOS does not permit references to inaccessible data. We require that authors provide all relevant data within the paper, Supporting Information files, or in an acceptable, public repository. Please add a citation to support this phrase or upload the data that corresponds with these findings to a stable repository (such as Figshare or Dryad) and provide and URLs, DOIs, or accession numbers that may be used to access these data. Or, if the data are not a core part of the research being presented in your study, we ask that you remove the phrase that refers to these data.

If the reviewer comments include a recommendation to cite specific previously published works, please review and evaluate these publications to determine whether they are relevant and should be cited. There is no requirement to cite these works unless the editor has indicated otherwise.

[Note: HTML markup is below. Please do not edit.]

Reviewers' comments:

Reviewer's Responses to Questions

**Comments to the Author**

1. Is the manuscript technically sound, and do the data support the conclusions?

Reviewer #1: Partly

Reviewer #2: Yes

**********

2. Has the statistical analysis been performed appropriately and rigorously?

Reviewer #1: Yes

Reviewer #2: Yes

**********

3. Have the authors made all data underlying the findings in their manuscript fully available?

The PLOS Data policy

Reviewer #1: Yes

Reviewer #2: Yes

**********

4. Is the manuscript presented in an intelligible fashion and written in standard English?

Reviewer #1: Yes

Reviewer #2: Yes

**********

Reviewer #1: This manuscript examines the impact of early embryonic exposure to the SSRI paroxetine on neurodevelopment

using a zebrafish model. The authors report that paroxetine triggers early apoptosis, alters neurogenesis, and leads

to later changes in brain morphology and retinal projections, which are interpreted as brain-preferential remodeling.

The study also look at how these effects compare to 5-HT exposure to explore potential underlying mechanisms. The

study addresses an important question regarding the direct effects of SSRI exposure during development and offers

a dataset that integrates imaging, genetic reporters, and behavioral assays. The work is generally well-executed and

suitable for PLOS ONE, but some parts of how the data is interpreted and how clear it is need to be changed.

Major comments:

1. The manuscript does not clearly distinguish whether the observed phenotypes arise from decreased intracellular

serotonin (due to SERT inhibition) or increased extracellular serotonin signaling. The differential effects of paroxetine

and 5-HT (e.g., apoptosis observed only with paroxetine, but neurogenesis defects seen with both) are not sufficiently

explained. A clearer and more cautious interpretation of these mechanistic differences is needed.

2. Figure 1 presents sequence and structural conservation of SERT between human and zebrafish. However, no

functional validation of paroxetine on zebrafish SERT is provided. The authors should acknowledge this limitation.

3. The increase in proliferating (pH3-positive) cells alongside reduced neuronal differentiation suggests altered

progenitor dynamics, but the underlying mechanism is unclear. It is not evident whether this reflects accumulation of

progenitor populations or impaired neuronal differentiation.

4. The interpretation of increased retinal projection area as evidence of “brain remodeling” appears overstated and

should be toned down. The analysis of retinotectal projections is primarily based on fluorescence area and intensity,

which does not distinguish between functional circuit restoration and aberrant axonal growth. The observed

expansion of retinal projections at later stages could reflect miswiring or impaired pruning rather than true remodeling.

I suggest the authors to revise the language and present these findings more cautiously as alterations in projection

patterns rather than definitive remodeling.

5. The observation that 5-HT phenocopies certain aspects of the paroxetine-induced phenotype (e.g., altered

neurogenesis and projection changes) is not mechanistically explained. The authors should clarify whether 5-HT is

being used as a control, or comparator, and adjust their interpretation accordingly.

Minor comments:

1. The use of terms such as “restoration” should be reconsidered and replaced with more neutral descriptions.

2. What links SERT inhibition to apoptosis? A brief discussion on this would be informative.

3. Given the known sex- specific differences in neurodevelopmental disorders such as ASD, it would be useful for

the authors to briefly discuss whether sex-specific effects could influence their findings. While this may not be feasible

to assess at early developmental stages in zebrafish, acknowledging this as a limitation would strengthen the

manuscript.

4. The manuscript would benefit from general language editing to correct grammatical errors and improve clarity (e.g.,

typographical errors such as “occurrs” (line no.165) and inconsistent terminology (e.g., “parox” vs “paroxetine))

Reviewer #2: The manuscript “Paroxetine-induced transient apoptosis with delayed neurogenesis induces brain remodeling in developing zebrafish" by Sato and colleagues presents an interesting investigation by demonstrating that early exposure of zebrafish embryos to paroxetine retards neurogenesis in the optic tectum and the telencephalon.

The work is technically solid, the experimental execution sound, and the results show that paroxetine-treated embryos exhibit impaired growth, with small heads and short body lengths resulting from transient apoptosis in the animal model.

The article is very impressive, contributing to a deeper understanding of the biological mechanisms underlying early exposure to paroxetine, which, as the authors argue, constitutes a risk factor implicated in the pathogenesis of autism spectrum disorder (ASD).

The topic is timely and relevant to neurodevelopmental disorders and the use of zebrafish to study these conditions. The manuscript introduces fundamentally new mechanisms that would significantly advance current understanding.

The quantity of results presented in the main article and in the supplementary material is commendable. The discussion, in turn, clearly presents the experimental findings and thoughtfully situates them within the context of existing literature. This approach effectively demonstrates consistency with prior studies and reinforces the reliability of the results. Besides, careful comparison with previous work contributes to a coherent narrative and supports the manuscript's scientific relevance, thereby enhancing its overall clarity and scholarly value.

Lastly, starting on pg. 90, the authors included their responses to the Reviewers’, clearly demonstrating that all suggestions in the previous round have been carefully addressed. They explain that it took longer than six months to revise and address reviewers' comments. This thorough and transparent response reflects the authors’ commitment to improving the manuscript and strengthens the work's overall quality and rigor.

In the opinion of this referee, the only remaining question raised by referees is the overgeneralization of results to humans. Therefore, I recommend that the authors re-revise their work to interpret their findings and draw conclusions that are supported by their data.

**********

what does this mean?). If published, this will include your full peer review and any attached files.

If you choose “no”, your identity will remain anonymous but your review may still be made public.

**Do you want your identity to be public for this peer review?**  For information about this choice, including consent withdrawal, please see our Privacy Policy

Reviewer #1: No

Reviewer #2: No

**********

[NOTE: If reviewer comments were submitted as an attachment file, they will be attached to this email and accessible via the submission site. Please log into your account, locate the manuscript record, and check for the action link "View Attachments". If this link does not appear, there are no attachment files.]

To ensure your figures meet our technical requirements, please review our figure guidelines: https://journals.plos.org/plosone/s/figures

You may also use PLOS’s free figure tool, NAAS, to help you prepare publication quality figures: https://journals.plos.org/plosone/s/figures#loc-tools-for-figure-preparation.

NAAS will assess whether your figures meet our technical requirements by comparing each figure against our figure specifications.

# Author comment: Author response to Decision Letter 1

**DOI:** 10.1371/journal.pone.0345903.r003

18 Jul 2026

Response Letter to Reviewers

PONE-D-26-05926

Paroxetine-induced transient apoptosis with delayed neurogenesis triggers brain-preferential regeneration in developing zebrafish

Tomomi Sato

PLOS One

Summary of Responses to the Reviewers’ comments:

Responses to the Reviewers’ each comment

Reviewer #1: This manuscript examines the impact of early embryonic exposure to the SSRI paroxetine on neurodevelopment using a zebrafish model. The authors report that paroxetine triggers early apoptosis, alters neurogenesis, and leads to later changes in brain morphology and retinal projections, which are interpreted as brain-preferential remodeling. The study also looks at how these effects compare to 5-HT exposure to explore potential underlying mechanisms. The study addresses an important question regarding the direct effects of SSRI exposure during development and offers a dataset that integrates imaging, genetic reporters, and behavioral assays. The work is generally well-executed and suitable for PLOS ONE, but some parts of how the data is interpreted and how clear it is need to be changed.

Major comments

Comment 1: The manuscript does not clearly distinguish whether the observed phenotypes arise from decreased intracellular serotonin (due to SERT inhibition) or increased extracellular serotonin signaling. The differential effects of paroxetine and 5-HT (e.g., apoptosis observed only with paroxetine, but neurogenesis defects seen with both) are not sufficiently explained. A clearer and more cautious interpretation of these mechanistic differences is needed.

Our answer: Thank you for the reviewer’s critical comments. We interpret our results to mean that the apoptosis only induced by paroxetine is due to decreased intracellular serotonin by SERT inhibition, whereas the decreased neurogenesis observed both in paroxetine and 5-HT is primarily caused by the apoptosis for paroxetine as described in the Results “suggesting that increased apoptosis induced by paroxetine reduced the number of post-mitotic neurons (Fig 3B, D-F)” (lines 382–383). However, the decreased neurogenesis is also observed in 5-HT without apoptosis induction, implying that it is potentially caused by neural stem/progenitor cell expansion due to increased extracellular serotonin for 5-HT. Consistently, we detected an increase in the number of pH3-positive mitotic cells in the SVZ (Fig. 4I, J) and SOX2 expression (S4K, L Fig). Therefore, according to the reviewer’s valuable comments, we added cautious interpretation of the differential effects of paroxetine and 5-HT in the Results as follows: “This observation implied that reduced neurogenesis can potentially result from expansion of neural stem/progenitor cells.” (lines 393–394) and “these results suggest that paroxetine secondarily promotes the proliferation of neural progenitor cells following apoptosis and consequently impairs neurogenesis in the optic tectum.” (lines 407–409). Furthermore, to define our interpretation more clearly, we added a sentence in the Discussion as follows: “The mechanisms underlying a transition from paroxetine-induced transient apoptosis to increased mitotic cells in the SVZ remain to be clarified.” (lines 609–611). We also removed phrases from the Discussion as follows: “This may provide insight into a biological basis for the neurodevelopmental disorders associated with early-onset FGR.” (lines 745–746, in a marked-up copy), and “and increased 5-HT” (line 689, in a marked-up copy).

Comment 2: Figure 1 presents sequence and structural conservation of SERT between human and zebrafish. However, no functional validation of paroxetine on zebrafish SERT is provided. The authors should acknowledge this limitation.

Our answer: According to the reviewer’s helpful comment, we added the description that “Although we did not functionally validate the effects of paroxetine on zebrafish SERT, we demonstrate strong sequence conservation of SERT between humans and zebrafish.” in Discussion (lines 438–440).

Comment 3: The increase in proliferating (pH3-positive) cells alongside reduced neuronal differentiation suggests altered progenitor dynamics, but the underlying mechanism is unclear. It is not evident whether this reflects accumulation of progenitor populations or impaired neuronal differentiation.

Our answer: We showed that paroxetine caused an increase in proliferating (pH3-positive) cells in the SVZ (Fig 4I, L), concomitant with reduced neuronal differentiation (Fig 4E, F) and enhanced expression of SOX2 that promotes self-renewal of neural progenitor cells (S4K¬¬, L Fig). These results suggest that accumulation of progenitor populations consequently leads to impaired neuronal differentiation. In addition, those results explain the reason why GFP-positive neurons are subsequently restored at later stages in the optic tectum and the telencephalon (Fig 5F, ¬H and Fig 6F, H) . To make the underlying mechanism clear, we deleted the phrase “suppress cell differentiation and” (line 443) and changed the description to “these results suggest that paroxetine secondarily promotes the proliferation of neural progenitor cells following apoptosis and consequently impairs neurogenesis in the optic tectum.” (lines 268–270).

Comment 4: The interpretation of increased retinal projection area as evidence of “brain remodeling” appears overstated and should be toned down. The analysis of retinotectal projections is primarily based on fluorescence area and intensity, which does not distinguish between functional circuit restoration and aberrant axonal growth. The observed expansion of retinal projections at later stages could reflect miswiring or impaired pruning rather than true remodeling. I suggest the authors to revise the language and present these findings more cautiously as alterations in projection patterns rather than definitive remodeling.

Our answer: Thank you for the reviewer’s reasonable comments. According to the comments, we replaced the word “brain remodeling” in the title with “brain-preferential regeneration” (lines 2–3) and the phrase “suggesting brain-preferential remodeling” in the Abstract with “suggesting brain-preferential regeneration” (line 34). We changed the words “remodeling” to “regeneration” in the Abstract (line 32), the Results (lines 303, 460, 461, 503), the Fig 5 and 6 captions (line 470, 542, respectively) and the Discussion (lines 628, 642, 649). In addition, the word “remodeling” in the Discussion was replaced with “reorganization” (lines 632 and 650) and the words “remodeling period” in the Discussion with “this period” (line 633).

Comment 5: The observation that 5-HT phenocopies certain aspects of the paroxetine-induced phenotype (e.g., altered neurogenesis and projection changes) is not mechanistically explained. The authors should clarify whether 5-HT is being used as a control, or comparator, and adjust their interpretation accordingly.

Our answer: We appreciate the reviewer’s insightful comments. We used 5-HT as a comparator in order to segregate the direct from the indirect effects of paroxetine, that is, the SERT-inhibiting effect from the resulting effect of increased extracellular 5-HT levels. Because, those effects are supposed to be the underlying mechanisms for the therapeutic effect of SSRIs including paroxetine as antidepressants. This purpose was added in the Introduction as follows: “In this study, we used 5-HT as a comparator of paroxetine in order to segregate the direct from the indirect effects of paroxetine, that is, the SERT-inhibiting effect from the resulting increased extracellular 5-HT levels.” (lines 86–89). By comparing paroxetine with 5-HT, we investigated which and how effects of paroxetine act on brain development. Unexpectedly, we found that both the treatment with paroxetine and 5-HT had similar effects on neurogenesis and projection. Our interpretation of those results has been described in the Discussion as follows: “These findings suggest that decreased neurogenesis may contribute to the extension of retinal axons, as this phenotype occurs irrespective of whether growth restriction and localized apoptosis are induced.” (lines 639–641). Because their molecular mechanisms remain unknown, we added the description as follows: “However, the underlying mechanisms by which decreased neurogenesis leads to extended axonal projections remain to be defined.” (lines 641–642).

Minor comments

Comment 1: The use of terms such as “restoration” should be reconsidered and replaced with more neutral descriptions.

Our answer: According to the reviewer’s comment, we replaced the word “restoration” with more neutral descriptions such as “regrowth” or “regeneration” depending on the context.

Comment 2: What links SERT inhibition to apoptosis? A brief discussion on this would be informative.

Our answer: According to the reviewer’s helpful comment, we added a brief discussion on the potential mechanisms of paroxetine-induced apoptosis as follows: In rat glioma and human neuroblastoma cell lines, paroxetine and fluoxetine have been reported to cause apoptosis through a rapid increase in p-c-Jun, Cytochrome c release from mitochondria and increased Caspase-3 activity [51]. These data suggest a potential mechanism underlying paroxetine-induced apoptosis in the brain. (lines 617–621).

Comment 3: Given the known sex- specific differences in neurodevelopmental disorders such as ASD, it would be useful for the authors to briefly discuss whether sex-specific effects could influence their findings. While this may not be feasible to assess at early developmental stages in zebrafish, acknowledging this as a limitation would strengthen the manuscript.

Our answer: We appreciate useful comments from the reviewer. Although we understand the importance of sex-specific differences in neurodevelopmental disorders such as ASD, such sex-specific effects are not considered in this study because of the difficulty of identifying those effects at early zebrafish embryos. Therefore, we acknowledged this as a limitation in the Discussion as follows: “However, the interactions between these effects and other risk factors, including sex-specific influences, remain unresolved.” (lines 679-680).

Comment 4: The manuscript would benefit from general language editing to correct grammatical errors and improve clarity (e.g., typographical errors such as “occurrs” (line no.165) and inconsistent terminology (e.g., “parox” vs “paroxetine)).

Our answer: According to the reviewer’s comment, we corrected for the typographical errors such as “occurrs” with “occurs” and for the inconsistent terminology including “parox” with consistently use the term “paroxetine” in the main text. We also utilized English proofreading by ENAGO to correct grammatical errors in our revised manuscript. Our corrections are indicated in light blue, those in response to the reviewer’s comments in light green, and those according to ENAGO in red in a marked-up copy of the revised manuscript.

Reviewer #2:

The manuscript “Paroxetine-induced transient apoptosis with delayed neurogenesis induces brain remodeling in developing zebrafish" by Sato and colleagues presents an interesting investigation by demonstrating that early exposure of zebrafish embryos to paroxetine retards neurogenesis in the optic tectum and the telencephalon.

The work is technically solid, the experimental execution sound, and the results show that paroxetine-treated embryos exhibit impaired growth, with small heads and short body lengths resulting from transient apoptosis in the animal model.

The article is very impressive, contributing to a deeper understanding of the biological mechanisms underlying early exposure to paroxetine, which, as the authors argue, constitutes a risk factor implicated in the pathogenesis of autism spectrum disorder (ASD).

The topic is timely and relevant to neurodevelopmental disorders and the use of zebrafish to study these conditions. The manuscript introduces fundamentally new mechanisms that would significantly advance current understanding.

The quantity of results presented in the main article and in the supplementary material is commendable. The discussion, in turn, clearly presents the experimental findings and thoughtfully situates them within the context of existing literature. This approach effectively demonstrates consistency with prior studies and reinforces the reliability of the results. Besides, careful comparison with previous work contributes to a coherent narrative and supports the manuscript's scientific relevance, thereby enhancing its overall clarity and scholarly value.

Lastly, starting on pg. 90, the authors included their responses to the Reviewers’, clearly demonstrating that all suggestions in the previous round have been carefully addressed. They explain that it took longer than six months to revise and address reviewers' comments. This thorough and transparent response reflects the authors’ commitment to improving the manuscript and strengthens the work's overall quality and rigor.

In the opinion of this referee, the only remaining question raised by referees is the overgeneralization of results to humans.

Therefore, I recommend that the authors re-revise their work to interpret their findings and draw conclusions that are supported by their data.

Our answer:

Thank you for the reviewer’s critical comments. Because we had obtained new insights from this study into another project exploring early biomarkers of maternal depression, we described much speculation about generalization to humans. According to the reviewer’s comments, we deleted most of the description of the overgeneralization of results to humans from the Discussion, such as the description of fluvoxamine in COVID-19 deterioration, thalidomide teratogenic effects in offspring, and maternal inflammation through DAMPs. We also deleted any speculation from schematic in S8 Fig. However, we left the description of FGR and associations with ASD in humans and translocated those descriptions to the last part of the paragraph in the Discussion. Next, we simplified the Discussion by eliminating redundancy and rearranged it into four paragraphs as follows:

1) Paroxetine-induced FGR

2) Paroxetine-induced apoptosis

3) Proxetine/5-HT-induced delayed neurogenesis

4) Paroxetine/5-HT-triggered impact on social behavior

Attachment Submitted filename: Response Letter2_20260719.docx

# Peer review: Decision Letter 1

**DOI:** 10.1371/journal.pone.0345903.r004

**Authors:** Suresh Yenugu

3 Sep 2026

Paroxetine-induced transient apoptosis with delayed neurogenesis triggers brain-preferential regeneration in developing zebrafish

PONE-D-26-05926R1

Dear Dr. Sato,

We’re pleased to inform you that your manuscript has been judged scientifically suitable for publication and will be formally accepted for publication once it meets all outstanding technical requirements.

Within one week, you’ll receive an e-mail detailing the required amendments. When these have been addressed, you’ll receive a formal acceptance letter and your manuscript will be scheduled for publication.

An invoice will be generated when your article is formally accepted. Please note, if your institution has a publishing partnership with PLOS and your article meets the relevant criteria, all or part of your publication costs will be covered. Please make sure your user information is up-to-date by logging into Editorial Manager at Editorial Manager® and clicking the ‘Update My Information' link at the top of the page. For questions related to billing, please contact billing support.

If your institution or institutions have a press office, please notify them about your upcoming paper to help maximize its impact. If they’ll be preparing press materials, please inform our press team as soon as possible -- no later than 48 hours after receiving the formal acceptance. Your manuscript will remain under strict press embargo until 2 pm Eastern Time on the date of publication. For more information, please contact onepress@plos.org.

Kind regards,

Suresh Yenugu

Academic Editor

PLOS One

Additional Editor Comments (optional):

Reviewers' comments:

Reviewer's Responses to Questions

**Comments to the Author**

Reviewer #1: All comments have been addressed

Reviewer #2: All comments have been addressed

**********

2. Is the manuscript technically sound, and do the data support the conclusions?

Reviewer #1: Yes

Reviewer #2: Yes

**********

3. Has the statistical analysis been performed appropriately and rigorously?

Reviewer #1: I Don't Know

Reviewer #2: Yes

**********

4. Have the authors made all data underlying the findings in their manuscript fully available?

The PLOS Data policy

Reviewer #1: Yes

Reviewer #2: Yes

**********

5. Is the manuscript presented in an intelligible fashion and written in standard English?

Reviewer #1: Yes

Reviewer #2: Yes

**********

Reviewer #1: The authors have sufficiently addressed all the comments raised by me. I do not have any further comments on the revision.

Reviewer #2: The topic is timely and highly relevant to neurodevelopmental disorders and the use of zebrafish as a model system. The manuscript introduces novel mechanisms that significantly advance the current understanding in the field. The authors have fully addressed all reviewer comments, and I am satisfied with the revisions.

**********

what does this mean?). If published, this will include your full peer review and any attached files.

If you choose “no”, your identity will remain anonymous but your review may still be made public.

**Do you want your identity to be public for this peer review?**  For information about this choice, including consent withdrawal, please see our Privacy Policy

Reviewer #1: No

Reviewer #2: **Yes:**  Branco CS

**********

# Editor report: Acceptance letter

**DOI:** 10.1371/journal.pone.0345903.r005

**Authors:** Suresh Yenugu

PONE-D-26-05926R1

PLOS One

Dear Dr. Sato,

I'm pleased to inform you that your manuscript has been deemed suitable for publication in PLOS One. Congratulations! Your manuscript is now being handed over to our production team.

At this stage, our production department will prepare your paper for publication. This includes ensuring the following:

* All references, tables, and figures are properly cited

* All relevant supporting information is included in the manuscript submission,

* There are no issues that prevent the paper from being properly typeset

You will receive further instructions from the production team, including instructions on how to review your proof when it is ready. Please keep in mind that we are working through a large volume of accepted articles, so please give us a few days to review your paper and let you know the next and final steps.

Lastly, if your institution or institutions have a press office, please let them know about your upcoming paper now to help maximize its impact. If they'll be preparing press materials, please inform our press team within the next 48 hours. Your manuscript will remain under strict press embargo until 2 pm Eastern Time on the date of publication. For more information, please contact onepress@plos.org.

You will receive an invoice from PLOS for your publication fee after your manuscript has reached the completed accept phase. If you receive an email requesting payment before acceptance or for any other service, this may be a phishing scheme. Learn how to identify phishing emails and protect your accounts at https://explore.plos.org/phishing.

If we can help with anything else, please email us at customercare@plos.org.

Thank you for submitting your work to PLOS One and supporting open access.

Kind regards,

PLOS One Editorial Office Staff

on behalf of

Dr. Suresh Yenugu

Academic Editor

PLOS One
